# Marine diterpenoid targets STING palmitoylation in mammalian cells

**DOI:** 10.1038/s42004-023-00956-9

**Published:** 2023-07-18

**Authors:** Wan-Chi Hsiao, Guang-Hao Niu, Chen-Fu Lo, Jing-Ya Wang, Ya-Hui Chi, Wei-Cheng Huang, Chun-Wei Tung, Ping-Jyun Sung, Lun Kelvin Tsou, Mingzi M. Zhang

**Affiliations:** 1 Institute of Molecular and Genomic Medicine, National Health Research Institutes, Miaoli, 35053 Taiwan; 2 Institute of Biotechnology, National Tsing Hua University, Hsinchu, 30013 Taiwan; 3 Institute of Biotechnology and Pharmaceutical Research, National Health Research Institutes, Miaoli, 35053 Taiwan; 4 Department of Marine Biotechnology and Resources, National Sun Yat-sen University, Kaohsiung, 804201 Taiwan; 5 National Museum of Marine Biology and Aquarium, Pingtung, 944401 Taiwan; 6 Chinese Medicine Research and Development Center, China Medical University Hospital, Taichung, 404394 Taiwan; 7 Graduate Institute of Natural Products, Kaohsiung Medical University, Kaohsiung, 807378 Taiwan

**Keywords:** Target identification, Proteomics, Mass spectrometry, Target identification, Natural products

## Abstract

Natural products are important sources of therapeutic agents and useful drug discovery tools. The fused macrocycles and multiple stereocenters of briarane-type diterpenoids pose a major challenge to total synthesis and efforts to characterize their biological activities. Harnessing a scalable source of excavatolide B (excB) from cultured soft coral *Briareum stechei*, we generated analogs by late-stage diversification and performed structure-activity analysis, which was critical for the development of functional excB probes. We further used these probes in a chemoproteomic strategy to identify Stimulator of Interferon Genes (STING) as a direct target of excB in mammalian cells. We showed that the epoxylactone warhead of excB is required to covalently engage STING at its membrane-proximal Cys91, inhibiting STING palmitoylation and signaling. This study reveals a possible mechanism-of-action of excB, and expands the repertoire of covalent STING inhibitors.

## Abstract (web summary)

Excavatolide B (excB) is a marine briarane type diterpenoid with anti-inflammatory properties, however, its cellular targets and mode-of-action remain unknown. Here, the authors develop two covalent probes of excB and apply them through a chemoproteomics approach to identify STING as a direct target of excB in living mammalian cells.

## Introduction

Natural products from microbes, plants and animals are important sources of therapeutic agents, including a large proportion of current drugs and recently approved new chemical entities^1,2^. Evolutionarily selected to interact with biomolecules, natural products offer opportunities to explore biologically-relevant regions of chemical space that may not be readily explored by synthetic small molecules^3^, and provide excellent starting points to address challenging protein targets such as K-Ras and Keap1^1,4,5^. Moreover, the identification of cellular targets and binding pockets of natural products have facilitated the discovery of unique druggable modalities such as small-molecule protein degraders^6–9^. Therefore, natural products are increasingly valued as drug discovery tools.

Corals are a rich source of marine natural products with diverse carbon scaffolds^10^, including briarane-type diterpenoids with their characteristic [8.4.0] bicyclic tetradecane core structures that often possess a γ-lactone spanning C7 and C8^11–13^. Exclusively isolated from gorgonian octocorals, the >700 briarane-type diterpenoids constitute one of the most common natural product families from these marine invertebrates and exhibit a myriad of anti-inflammatory, anti-microbial and anti-viral activities^11–15^. Yet for many briarane-type diterpenoids, in-depth studies of their biological activities are severely hampered by the lack of material and challenging chemical syntheses. No total synthesis of a briarane-type diterpenoid has been completed to date^16–18^. In addition, no direct cellular target of this class of natural products has been reported. Originally identified from the Formosan gorgonian coral *Briareum stechei* in 1998^19,20^, excavatolide B (excB) is one of the best characterized briarane-type diterpenoid in terms of its in vitro and in vivo biological activities, exhibiting promising anti-inflammatory properties with low cytotoxicity^19,21–23^. While we hypothesized excB to be an electrophilic natural product that exerts its biological effects by engaging target proteins via its epoxylactone warhead^24^, its cellular targets and mechanisms of action are unknown.

Here we describe the development and application of functional excB probes to identify the cellular targets of excB in mammalian cells. Our previously established methods for *B. stechei* mariculture and excB isolation ensured a stable supply of the natural product for late-stage modification, which enabled structure-activity relationship (SAR) analyses that guided our design of biologically active probes. Employing a chemoproteomic strategy with competition using natural and synthetic excB analogs, we showed Stimulator of Interferon Genes (STING) to be a direct cellular target of excB. Through in silico molecular docking, competition with known covalent STING inhibitors, biochemistry, genetics, and mass spectrometry, we demonstrated that excB specifically engaged STING at a membrane-proximal cysteine residue and inhibited STING *S*-palmitoylation through direct competition. Using cellular assays, we further showed that excB inhibited STING signaling as well as STING-mediated type I interferon (IFN) response in mammalian cells.

## Results

### Design and synthesis of functional excB probes

We observed that the naturally occurring briaexcavatolide L (BExcL)^25^, an epoxide ring-opened analog of excB, exhibited significantly reduced anti-inflammatory effects. BExcL did not inhibit inducible nitric oxide synthase (iNOS) gene expression and production of nitric oxide, and only marginally inhibited the production of inflammatory cytokine IL-6 production in LPS-treated RAW 264.7 macrophages (Supplementary Figs. 1–3). Given that the 8,17-epoxide of excB is critical for its anti-inflammatory activities, we hypothesized that excB engages its target proteins through covalent modification of nucleophilic amino acid residues. As a covalent natural product, excB should be readily amendable to chemoproteomic strategies for target identification.

**Fig. 1:**
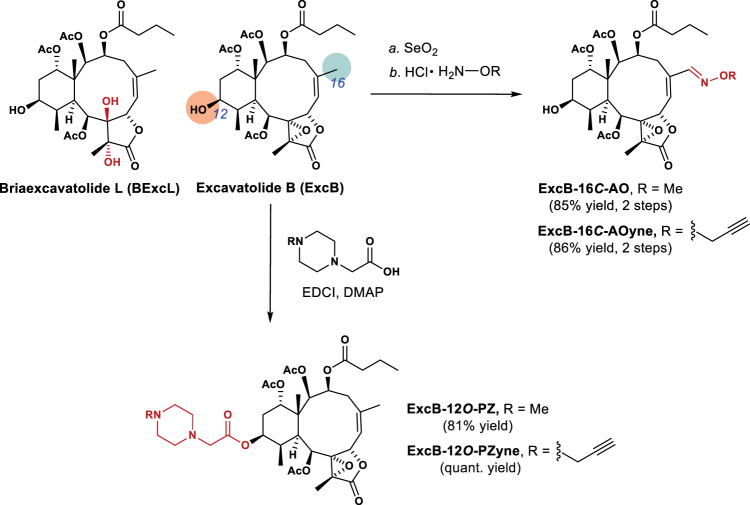
Synthesis of excB probes. Briarane-type diterpenoids BExcL and excB were isolated from the extracts of cultured *B. stechei*. Late-stage modification of excB at C12 and C16 was employed to generate excB analogs and probes used in this study. For details, see Supplementary Methods and Supplementary Data 1.

Towards that end, we synthesized clickable excB probes by appending a terminal alkyne on the briarane-type diterpenoid. The relatively small terminal alkyne moiety minimizes perturbation of excB’s chemical structure, which is critical for its target selectivity and biological activity. Introduction of the terminal alkyne also allows bioorthogonal conjugation of azide-containing tags by copper-catalyzed azide-alkyne cycloaddition (CuAAC) for fluorescent visualization and enrichment of excB targets. Since the unique 6,10,5-tricyclic skeleton with extensive stereoselective oxygenation of excB posed a formidable challenge for total synthesis, we leveraged our previously established methods for *B. stechei* mariculture and excB isolation to ensure a stable source of the natural product for derivatization via late-stage modification. Based on our SAR study that identified possible sites for excB modification^26^, we synthesized excB-12*O*-PZ bearing an *N*-methylpiperazine moiety using Steglich esterification on 12-OH as well as excB-16*C*-AO, which was obtained using Riley oxidation on C16 followed by condensation with hydroxylamine (Fig. 1). The observation that both excB-12*O*-PZ and excB-16*C*-AO retained anti-inflammatory activities prompted us to generate the respective alkynyl probes (Supplementary Figs. 1–3). Following similar strategies, excB-12*O*-PZyne and excB-16*C*-AOyne were synthesized in quantitative and 86% yields over 2 steps, respectively (Fig. 1). The two structurally distinct excB probes retained the anti-inflammatory properties of excB, demonstrating inhibition of *iNOS* expression as well as nitric oxide and IL-6 production in LPS-treated RAW 264.7 macrophages (Supplementary Figs. 1–3).

### ExcB covalently modifies proteins in living cells

**Fig. 2:**
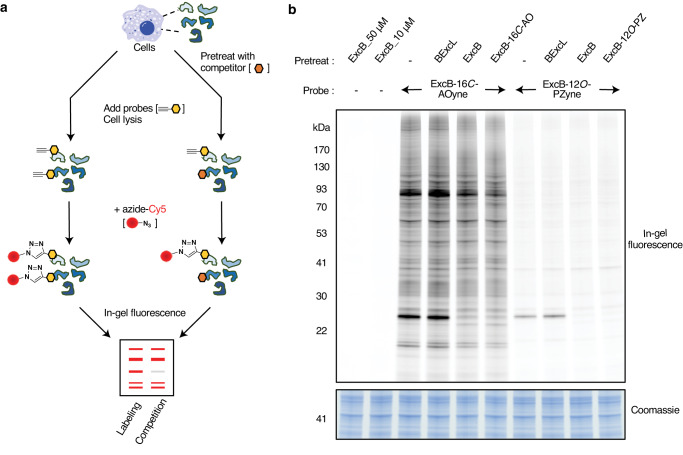
Fluorescent detection of excB targets in living cells. **a** Workflow for fluorescence detection of excB targets in mammalian cells using excB-16*C*-AOyne and excB-12*O*-PZyne with azide-Cy5. **b** RAW 264.7 cells were pretreated for 2 h with 40 μM of excB or its analogs followed by 1 h treatment with 10 μM of indicated probes. Coomassie stain acts as loading control for the accompanying fluorescence gel with indicated protein molecular weight markers. Data were representative of three independent biological repeats.

Having shown excB-12*O*-PZyne and excB-16*C*-AOyne to be functional probes, we next tested if they can be used to label and detect excB targets in living cells by in-gel fluorescence (Fig. 2a). Based on our SAR study, which showed that different modifications at C12 and C16 can have large effects on the anti-inflammatory activities of excB^26^, we expected the two structurally distinct probes to have potentially different specific and non-specific targets. Indeed, we observed differences in the target profiles of excB-12*O*-PZyne and excB-16*C*-AOyne, especially in the overall labeling intensity (Fig. 2b). Competition experiments revealed that both probes shared a subset of protein targets with excB. Pretreating cells with excB or functional excB analogs, excB-12*O*-PZ and excB-16*C*-AO, blocked probe labeling of specific proteins. In contrast, pretreatment with BExcL did not compete probe labeling of those proteins (Fig. 2b). Overall, these results demonstrated that the 8,17-epoxide of excB is required for target engagement and, to help rule out false-positive targets, both excB-12*O*-PZyne and excB-16*C*-AOyne will be used to capture and identify the cellular targets of excB.

### ExcB covalently targets STING in mammalian cells

**Fig. 3:**
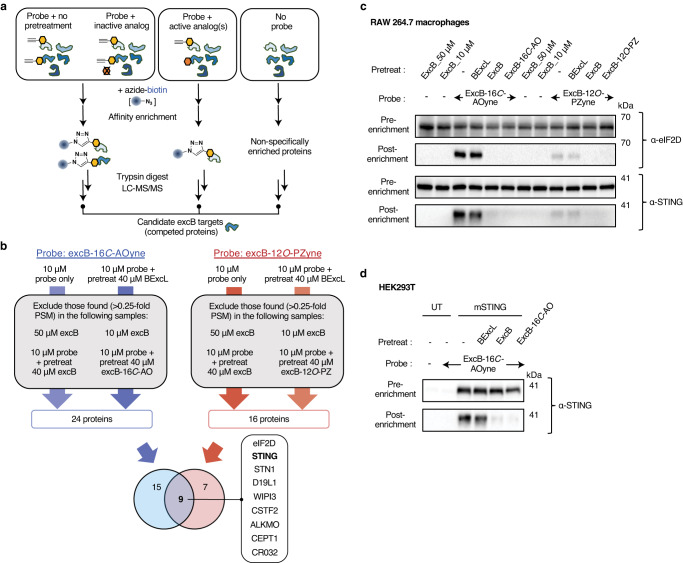
Chemoproteomics revealed STING as a target of excB. **a** Chemoproteomic strategy for the enrichment and identification of excB targets using excB probes. **b** Filter criteria used to identify excB targets using excB-16*C*-AOyne and excB-12*O*-PZyne from three independent biological repeats. Venn diagram represents overlap between high-confidence targets of the two probes (Supplementary Data 2). **c** RAW 264.7 macrophages were pretreated for 2 h with 40 μM of excB or indicated analogs prior to 1 h labeling with 10 μM excB-16*C*-AOyne or excB-12*O*-PZyne. Cell lysates were reacted with azide-biotin. Protein samples before and after NeutrAvidin enrichment were immunoblotted for eIF2D or STING. Western blot data were representative of at least 3 independent biological repeats. **d** HEK293T cells expressing mSTING were pretreated with the indicated excB analogs for 2 h prior to labeling with 10 μM excB-16*C*-AOyne for 15 min. After reaction with azide-biotin, protein samples before and after NeutrAvidin enrichment were immunoblotted for STING. UT, untransfected.

Given that excB likely exerts its biological function through covalent engagement of proteins, we sought to identify its cellular targets via a chemoproteomic strategy. We reasoned that probe labeling of functional excB targets will be competed away by excess excB and their respective biologically active analogs excB-12*O*-PZ and excB-16*C*-AO, but not by the inactive analog BExcL (Fig. 3a). By employing two excB probes in which the terminal alkyne was introduced at distinct locations of excB, we sought to capture and identify pharmacologically relevant targets of excB.

To identify the protein targets of excB, we pretreated RAW 264.7 macrophages with excB or its analogs prior to probe labeling. ExcB-12*O*-PZyne and excB-16*C*-AOyne labeled proteins were subsequently enriched using azide-biotin and identified by mass spectrometry. For each probe, we obtained a list of candidates that were preferentially enriched in probe-labeled samples without pretreatment or pretreated with inactive analog, but absent in non-probe-labeled samples or probe-labeled samples pretreated with functional excB analogs. From three independent proteomics experiments, 24 and 16 candidate proteins were identified for excB-16*C*-AOyne and excB-12*O*-PZyne, respectively. Of these, 9 proteins were common to both probes (Fig. 3b, Supplementary Data 2). We validated the top two hits prioritized based on spectral counts, eIF2D (eukaryotic translation initiation factor 2D) and STING, as targets of excB. Probe labeling and subsequent affinity enrichment of both proteins from the lysates of RAW 264.7 macrophages were specifically competed away by pretreatment with excB and its biologically active analogs (Fig. 3c, Supplementary Fig. 4). Similar protein levels in the input samples excluded the possibility of different protein expression levels as a result of compound treatments. In contrast, pretreatment with BExcL did not affect affinity enrichment of eIF2D and STING, underscoring the importance of the 8,17-epoxide for target engagement by excB.

We chose to focus on STING due to its critical role in immunity and inflammation as a key component of the cGAS-STING pathway involved in sensing cytosolic DNA^27^, as well as its potential relevance to the anti-inflammatory effects of excB. We further validated STING to be a target of excB by expressing murine STING (mSTING) in HEK293T cells and monitoring excB-16*C*-AOyne engagement. Consistent with our observations in RAW 264.7 macrophages, probe labeling of mSTING was specifically competed by excB and excB-16*C*-AO but not by BExcL using both Western blot and in-gel fluorescence detection methods (Fig. 3d, Supplementary Fig. 5). With in-gel fluorescence, we noticed a protein species of a slightly higher molecular weight compared to the main mSTING band in samples pretreated with excB and excB-16*C*-AO (Supplementary Fig. 5). Given that this protein species was also observed in other in-gel fluorescence experiments where STING-associated fluorescence was effectively competed (*vide infra*), it is likely a co-migrating protein species that was unveiled when the main mSTING-associated signal was removed. Since it was only present in HEK293T cells expressing mSTING and not in untransfected cells, it may be a minor post-translationally modified mSTING species that was not apparent by Western blot or a protein that became available for probe targeting upon mSTING expression. We did not follow up on this co-migrating mSTING-dependent species since it could not be competed by excB (Supplementary Fig. 5), suggesting that it is likely a non-specific target of excB-16*C*-AOyne. Overall, these experiments demonstrated that mSTING is a direct cellular target of excB.

### ExcB covalently engages STING at Cys91

**Fig. 4:**
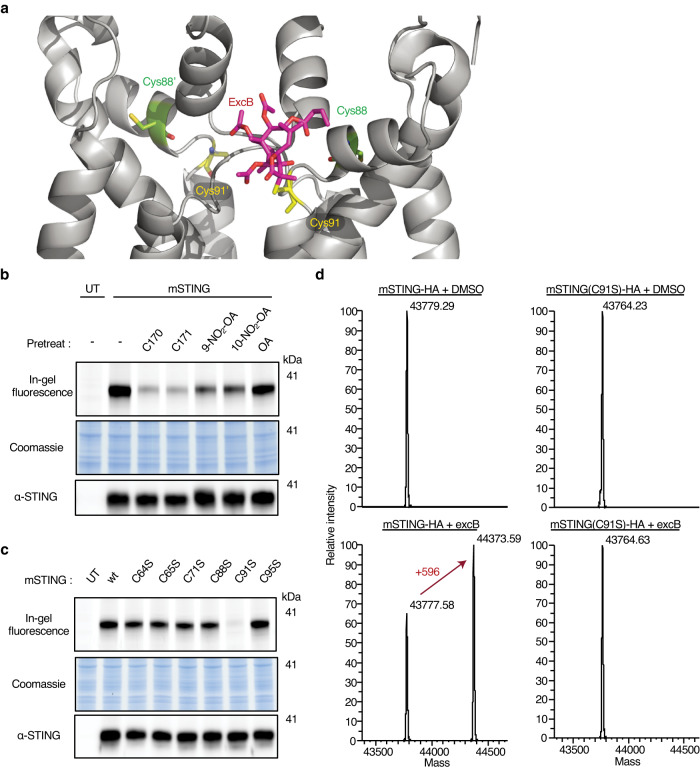
ExcB covalently engaged STING at membrane-proximal Cys91. **a** Covalent docking of excB (magenta) at Cys91 (yellow) of apo-hSTING dimer (6NT5). Cys88 (green) on both monomers are sterically unavailable for docking. **b** HEK293T cells expressing wild type (wt) mSTING were pretreated for 2 h with 10 μM of the indicated compounds prior to 15 min labeling with 10 μM excB-16*C*-AOyne. Probe labeling of mSTING was monitored by in-gel fluorescence. **c** HEK293T cells expressing wt mSTING and the indicated cysteine-to-serine mutants were labeled with 10 μM excB-16*C*-AOyne for 15 min. Probe labeling of mSTING was monitored by in-gel fluorescence. Anti-STING and Coomassie blue stain act as loading controls for the accompanying fluorescence gels. Western blot and in-gel fluorescence data were representative of 2 independent biological repeats. UT, untransfected. **d** Deconvoluted ESI mass spectra for HA-tagged mSTING and mSTING(C91S) immunopurified from HEK293T cells treated with DMSO or 10 μM excB for 2 h.

Having shown that STING is a cellular target of excB, we next sought to identify the nucleophilic site on STING engaged by excB. In silico docking revealed three potential binding pockets for excB on the surface of full-length apo-hSTING (human STING) dimer (Supplementary Fig. 6). Those were the cGAMP binding pocket, the binding pocket of STING agonist compound 53 in the transmembrane domain region^28^, and a membrane-proximal external region binding pocket. Compared to the first two binding pockets that were devoid of cysteines, we focused on the membrane-proximal external region binding pocket due to its proximity to a nucleophilic amino acid residue (Cys91) for excB engagement. In our model, a neighboring cysteine (Cys88) was buried in the structure and unavailable for excB engagement based on subsequent covalent docking (Fig. 4a, Supplementary Fig. 6). Together, our molecular docking results suggested that excB likely engages STING at Cys91.

We postulated that if excB covalently engages STING at Cys91, excB-16*C*-AOyne labeling of STING should be competitively blocked by STING inhibitors that are also known to covalently target Cys91. These inhibitors include C170, C171 and nitro-oleate^29,30^. As expected, pretreatment of mSTING-expressing HEK293T cells with C170, C171, and both regioisomers of nitro-oleate prominently diminished excB-16*O*-AOyne labeling of mSTING (Fig. 4b). In contrast, treatment with oleate did not affect mSTING labeling. We further showed that mSTING engagement by excB-16*C*-AOyne required Cys91 since mutation of Cys91, but not of other cysteines located in the N-terminal transmembrane domains of mSTING, drastically reduced probe labeling (Fig. 4c, Supplementary Fig. 7). Notably, while the adjacent Cys88 is known to be targeted by nitro-oleate^30^, this was not observed for excB since mutation of Cys88 did not affect excB-16*C*-AOyne labeling of mSTING. Given that STING modulators can have differential effects on mSTING and hSTING despite their high (>80%) amino acid similarity^28,29^, we further confirmed that excB also engaged hSTING mainly at Cys91 through competition with known covalent STING inhibitors and site-directed mutagenesis (Supplementary Fig. 8). No apparent cytotoxicity was observed for excB or its analogs in THP-1 macrophages. (Supplementary Fig. 9). Last but not least, we confirmed that the parent compound excB covalently engaged mSTING at the membrane-proximal Cys91 through intact mass analyses of mSTING and its C91S mutant. For mSTING-HA immunopurified from cells treated with excB, we observed a second peak with a mass difference of 596 Da, which corresponded to the molecular weight of excB (Fig. 4d). This second peak was not observed for mSTING(C91S)-HA immunopurified from excB-treated cells (Fig. 4d). Overall, these results supported our molecular docking study that excB covalently engages STING at Cys91.

### ExcB inhibits STING palmitoylation and signaling

**Fig. 5:**
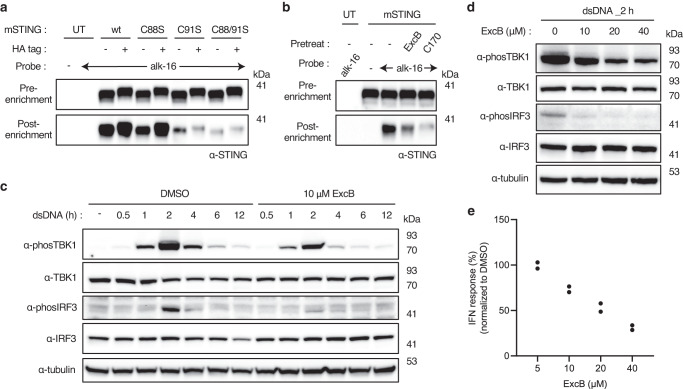
ExcB inhibited STING palmitoylation and signaling. **a** HEK293T cells expressing wild type (wt) mSTING or indicated cysteine mutants with or without HA tags were labeled with 50 μM alk-16 for 2 h. **b** HEK293T cells overexpressing mSTING were pretreated with 10 μM of the indicated inhibitors for 1.5 h prior to metabolic labeling with 50 μM alk-16 for 3.5 h. After reaction with azide-biotin, samples before and after NeutrAvidin enrichment were immunoblotted for STING. UT, untransfected. **c** Levels of total and phosphorylated TBK1 and IRF3 in NIH3T3 cells at indicated times after dsDNA-induced STING signaling. Cells were pretreated with DMSO or 10 μM excB for 1 h prior to dsDNA introduction. **d** Levels of total and phosphorylated TBK1 and IRF3 in NIH3T3 cells pretreated for 1 h with indicated concentrations of excB followed by dsDNA induction of STING signaling for 2 h. Equal volume of DMSO was used for 0 μM. **e** THP-1 monocytes containing the IRF-luciferase reporter were pretreated for 2.5 h with indicated concentrations of excB prior to cGAMP addition. IRF reporter activity was normalized to DMSO-treated controls (100%). *n* = 2.

Since excB covalently targeted STING at Cys91, which is a known *S*-palmitoylation site^31^, there may be direct competition between excB and palmitoyl acyltransferases for the same cysteine. To test this, we monitored STING palmitoylation using the previously characterized alk-16 chemical reporter for protein palmitoylation^32–34^. Both Western blot and in-gel fluorescence detection methods revealed that alk-16 labeling of mSTING was drastically reduced in HEK293T cells expressing the C91S or C88/91S mutants compared to wild type mSTING (Fig. 5a, Supplementary Fig. 10a–c). It is likely that Cys91 is the main mSTING palmitoylation site in HEK293T cells since no major change in alk-16 labeling was observed with the C88S mutant. Using this system to monitor mSTING palmitoylation, we showed that both excB as well as STING inhibitor C170 decreased mSTING palmitoylation (Fig. 5b, Supplementary Fig. 10d), which was expected if there was direct competition between the covalent inhibitors and palmitoyl acyltransferases for Cys91.

Given that palmitoylation is required for STING activation and type I interferon response^31^, we proceeded to test if excB treatment impaired STING signaling and function. Indeed, excB inhibited endogenous dsDNA-induced mSTING signaling in NIH3T3 cells as observed by the reduced levels of phosphorylated TBK1 and IRF3 in a dose-dependent manner (Fig. 5c, d). Similarly, dose-dependent inhibition of cGAMP-induced IFN response by excB was observed in human THP-1 reporter monocytes expressing luciferase under the control of an ISG54 (IFN-stimulated gene) minimal promoter and five IFN-stimulated response elements (Fig. 5e).

Overall, these results showed that excB inhibited STING signaling and STING-mediated IFN response through the direct competition and inhibition of STING palmitoylation at Cys91.

## Discussion

Briarane-type diterpenoids represent a structurally diverse family of bioactive marine natural products characterized by their core bicyclo[8.4.0]tetradecane ring system with an embedded γ-lactone at C7 and C8. Despite the wide range of biological activities observed for briarane-type diterpenoids, their cellular targets and mechanisms of action remain elusive. Enabled by the development of functional excB probes and chemoproteomics, our study revealed that excB interacts covalently with cellular proteins and provided the first examples of direct cellular targets of a briarane-type diterpenoid. It remains to be determined if other bioactive briarane-type diterpenoids, especially those with the epoxylactone warhead, covalently engage their protein targets and how functionalization (hydroxyls, esters, epoxides, and halogens) of the bicyclo[8.4.0]tetradecane scaffold contributes to target selectivity and bioactivity.

Chemoproteomic platforms have emerged as powerful tools to identify the targets of covalent compounds in complex biological samples and to harness the potential of natural products in drug discovery^35,36^. Natural product probes generated by total synthesis or late-stage modification enabled the rapid target identification and validation of bioactive natural products including ingenol mebutate^37^, parthenolide^38,39^, as well as excB (this study). Nonetheless, chemical derivatization to incorporate bioorthogonal or affinity handles continues to be challenging, particularly for structurally complex natural products^37^. Potential alteration of target specificity and bioactivity of the natural product probe upon chemical modification of the parent compound adds to this challenge. This is consistent with our observations that the two structurally distinct excB probes, with the bioorthogonal handle placed on different locations on the excB core scaffold, exhibited different labeling patterns and intensities. To rule out false-positive protein targets captured by each probe and identify the pharmacologically relevant targets, we focused on proteins captured by both probes and at the same time, were differentially competed by biologically active and non-electrophilic inactive analogs.

A complementary strategy to the probe-centric chemoproteomic approach is competitive activity-based protein profiling (ABPP) with broadly reactive probes such as iodoacetamide-alkyne. Compared to the probe-centric approach, proteins identified by competitive ABPP are limited by the target scope of the probes used and may be biased towards higher abundance protein targets. Nonetheless, by circumventing the need for chemical derivatization of natural products, this approach is extremely useful and increasingly used to profile the targets of natural products for which biologically active probes are unavailable^35^. Global cysteine profiling using competitive ABPP has been successful in mapping the targets of natural products including manumycin polyketides, nimbolide^40^, non-ribosomal peptides^41^, gambogic acid and others^38,42^.

The identification of STING as the direct cellular target of excB offers initial mechanistic insights into its anti-inflammatory effects. Using semi-quantitative mass spectrometry based on spectral counting, we identified STING as one of the main targets of excB but not any proteins with molecular weight ~25 kDa, which appeared in in-gel fluorescence experiments. We hypothesized that the ~25 kDa species may be a STING fragment or isoform since the excB-16*C*-AOyne associated fluorescence could be selectively and efficiently competed by different STING-specific covalent inhibitors with low background proteome reactivity (Supplementary Fig. 11)^29^. While we focused on STING in this study given its key role in innate immunity and the established anti-inflammatory activity of excB, we demonstrated that excB also covalently engaged eIF2D. It remains to be determined if and how the latter interaction contributes to the biological effects of excB since eIF2D-deficient mice are viable and free of apparent developmental defects (International Mouse Phenotyping Consortium). In addition, the role of eIF2D in translation initiation is still uncertain^43,44^. We also cannot exclude the possibility that excB has alternative targets in other tissues or cell types, including immune cells beyond macrophages.

Besides excB, the only other natural product reported to target STING is a plant-derived cyclopeptide astin C, which inhibits STING through a distinct mechanism. Astin C competitively binds to the C-terminal activation pocket of STING^45^, whereas excB selectively engages STING at Cys91 near its N-terminal transmembrane domain to block STING palmitoylation. Palmitoylation at membrane-proximal cysteine residues Cys88/91 is important for STING activation^31^, and can be targeted to inhibit STING signaling. Notably, Cys88/91 are hotspots for reported covalent STING inhibitors including nitrofurans (C170, C171, C178, C179)^29^, indole urea (H151)^29^, nitro-fatty acids^30^, and acrylamides (BPK-21, BPK-25)^46^. With its epoxylactone warhead, excB represents a new class of covalent STING inhibitors and, while unlikely to become a therapeutic in its current state, may inform the future design of STING modulators for treatment of inflammatory diseases^27^.

### Conclusion

A number of marine diterpenoids of the briarane family exhibit anti-inflammatory activities through unknown mechanisms of action and targets. Here we developed two structurally distinct excB probes and successfully employed them to identify STING as a direct target of excB in living mammalian cells. The epoxylactone warhead of excB is required for specific engagement of STING and its ability to inhibit STING signaling through direct competition of STING palmitoylation at Cys91. Our study identified a new class of covalent STING inhibitor and provide initial mechanistic insights into the anti-inflammatory activity of the briarane-type diterpenoid excB.

## Methods

### Chemicals and probes

All chemicals and reagents were purchased from Sigma-Aldrich unless otherwise indicated. Diazo-biotin-azide (CCR-1041) was purchased from DC Biosciences. Oleic acid (90260), 9-NO_2_-OA (10008042) and 10-NO_2_-OA (10008043) were purchased from Cayman Chemicals. C170 (HY-138682) and C171 (HY-138683) were purchased from MedChemExpress. ExcB was isolated from cultured *B. stechei* (see below) and BExcL was obtained from a previous study^25^. Synthetic excB analogs and probes as well as azide-Cy5 were synthesized in-house. For detailed chemical synthesis and characterization, see Supplementary Method and Supplementary Data 1.

### Animal material

Specimens of octocoral *B. stechei* in this study were initially collected in 2018 from Kenting ocean, Pingtung, Taiwan, and cultivated in an 80-ton culture reservoir containing a flowing seawater filtration system at the National Museum of Marine Biology & Aquarium (NMMBA), Taiwan (Supplementary Fig. 12). The sample was identified based on its morphology and micrographs of the coral sclerites, which were compared to a previous study^20,47^. A voucher specimen was deposited in the NMMBA (NMMBA-TW-SC-2018-052).

### Isolation of excB from *B. stechei*

Maricultural *B. stechei* specimens (wet weight 3.6 kg) were collected and minced into small pieces and lyophilized. Dry material (dry weight 1.9 kg) was milled and extracted with ethyl acetate (EtOAc) at room temperature to give EtOAc extract. The residue material was extracted again with 95% ethanol (EtOH) at room temperature to give EtOH extract. The EtOH extract was subjected to partition to afford a dichloromethane (CH_2_Cl_2_) phase. The EtOAc extract and CH_2_Cl_2_ phase were combined as the final crude extract (96.1 g). The crude extract was subjected to normal phase CC (silica gel, 70–230 mesh) with a gradient solvent system of hexanes/EtOAc/MeOH mixtures (100% hexanes, hexanes/EtOAc 90:10, 80:20, 70:30, 60:40, 50:50, 40:60, 30:70, 20:80, 10:90 (v/v), pure EtOAc, EtOAc/MeOH 80:20, 60:40, 50:50, 40:60, 20:80 (v/v), and pure MeOH) to obtain 17 subfractions (BS1–BS17). BS3 ~ BS8 (28.98 g) were combined based on TLC monitoring and further subjected to normal phase CC (silica gel, 230–400 mesh) with a gradient solvent system of hexanes/EtOAc mixtures (100% hexanes to pure EtOAc stepwise). ExcB (16.2 g) was recrystallized from the fraction of eluted hexanes/EtOAc 3:1. The overall yield of excB was 0.45% of *B. stechei* wet weight (16.2/3600 = 0.45%), which was approximately 4.5 g/kg of fresh coral.

### General analytical procedures for excB

1D and 2D NMR spectra were recorded on an ECZ-400 spectrometer (Jeol Ltd., Tokyo, Japan). The signals of the solution CDCl_3_ (residual CHCl_3_ (*δ*_H_ 7.26 ppm) and CDCl_3_ (*δ*_C_ 77.0 ppm)) were used as internal standards. NMR data were acquired and processed with Delta 6.1.0 or MestReNova v12.2.2 software. MS and HRMS spectra were acquired on an SolariX FTMS mass spectrometer (7 Tesla; Bruker, Bremen, Germany) with an ESI ion source in positive ionization mode. Silica gel (Kieselgel 60, 70–230 or 230–400 mesh, Merck, Darmstadt, Germany), and Sephadex LH-20 gel (Pharmacia Fine Chemicals AB, Uppsala, Sweden) were used for column chromatography (CC). Thin-layer chromatography (TLC) was carried out using silica gel (Kieselgel 60 F_254_, Merck) and RP-C18 (F_254s_, Merck) pre-coated plates. For visualization, the compounds were detected on TLC with a developer (50% H_2_SO_4_ (v/v) in methanol) followed by heating at 120 °C. HPLC analyses were performed with a pump (model L-2130, Hitachi, Tokyo, Japan) and a diode-array detector (model L-2455, Hitachi, Tokyo, Japan) equipped with an HPLC analytical column (BDS Hypersil^TM^ C18, 250 × 4.6 mm, 5 μm, ThermoFisher Scientific Inc., Waltham, MA, USA), and using a mixture of acetonitrile (ACN)/H_2_O or a mixture of methanol (MeOH)/H_2_O as the mobile phase (Supplementary Fig. 13).

### Cell lines and culture conditions

RAW 264.7, HEK293T and NIH3T3 cells were maintained in Dulbecco’s high glucose modified Eagle’s medium (DMEM, HyClone) supplemented with 10% (v/v) heat-inactivated fetal bovine serum (FBS, Biological Industries), penicillin (100 U/mL) and streptomycin (100 μg/mL). All cell lines were grown at 37 °C in a humidified 5% CO_2_ atmosphere. All cell lines were tested and confirmed to be mycoplasma-free using the MycoAlert® PLUS Mycoplasma Detection kit (Lonza, LT07-710).

### Fluorescent detection of excB protein targets

RAW 264.7 cells or STING-expressing HEK293T cells at 80–90% confluence in 6-well plates were treated with 10 μM of excB probes or equal volume of DMSO as control for 15–60 min. For competition experiments, cells were preincubated with 40 μM of excB or excB analogs for 2 h prior to probe addition. For STING inhibitor treatments, 10 μM of STING inhibitors (nitro-oleate, C170, C171) were added for 2 h prior to probe labeling. After probe labeling, cells were washed thrice with ice-cold PBS and pelleted at 400 × *g* for 5 min. Cells were flash-frozen in liquid nitrogen and stored at −80 °C prior to lysis. Frozen cells were lysed in SDS lysis buffer [4% SDS, 150 mM NaCl, 50 mM triethanolamine, pH 7.4, 2× EDTA-free protease inhibitor cocktail (ThermoFisher Scientific, 78439), 10 mM phenylmethylsulfonyl fluoride, 50 U/mL SuperNuclease (Sino Biological, SSNP01)]. Protein concentrations were determined by BCA protein assay (ThermoFisher Scientific, 23225). For in-gel fluorescence detection, 50 μg cell lysates were reacted with freshly made CuAAC reaction cocktail [100 μM azide-Cy5, 1 mM CuSO_4_, 1 mM tris(2-carboxyethyl) phosphine hydrochloride (TCEP), 100 μM tris[(1-benzyl-1H-1,2,3-triazol-4-yl)methyl]amine (TBTA)] in a total reaction volume of 50 μL for 1 h at room temperature. Proteins were chloroform-methanol precipitated and the pellet was washed twice with ice-cold methanol. Air-dried protein pellets were resuspended in 25 μL SDS buffer before the addition of 8.7 μL 4× SDS-loading buffer (20% glycerol, 125 mM Tris·HCl, pH 6.8, 4% SDS, 0.05% bromophenol blue) and 1.3 μL TCEP (ThermoFisher Scientific, 77720). Samples were heated at 95 °C for 5 min, separated by SDS-PAGE, and imaged on a ChemiDoc MP Imaging System (Bio-Rad). Cy5-associated signal was detected at excitation 625–650 nm/emission 675–725 nm. After fluorescence scanning, gels were either stained with Coomassie (Protein Ark, GEN-QC-STAIN) or transferred to PVDF membranes for Western blot analyses.

### Affinity enrichment of excB protein targets

For affinity purification of probe-labeled proteins from RAW 264.7 macrophages, 400 μg of cell lysates were diluted with HEPES buffer (150 mM NaCl, 50 mM HEPES pH 7.4) to 360 μL, and 40 μL of freshly prepared CuAAC reaction cocktail [diazo-biotin-azide (4 μL, 10 mM stock solution in DMSO), CuSO_4_ (8 μL, 50 mM aqueous solution), TCEP (8 μL, 50 mM aqueous solution), TBTA (20 μL, 2 mM stock solution in DMSO)] was added. After 1 h at room temperature, EDTA (8 μL of 0.5 M solution) was added and samples were chloroform-methanol precipitated. Air-dried protein pellets were resuspended in 80 μL SDS-HEPES buffer (4% SDS, 150 mM NaCl, 1 mM EDTA, 50 mM HEPES pH 7.4) and diluted to 0.5 mg/mL with HEPES buffer. The protein samples were then added to 25 μL of high-capacity NeutrAvidin beads (ThermoFisher Scientific, 29200), and incubated with end-over-end rotation for 90 min at room temperature. The beads were sequentially washed with 1 mL of 1% SDS in PBS (3 × 5 min), 4 M urea in PBS (2 × 5 min) and AMBIC (50 mM ammonium bicarbonate) (5 × 2 min).

For affinity purification and Western blot detection of probe-labeled STING from STING-expressing HEK293T cells, 50 μg of cell lysates in 45 μL SDS lysis buffer was reacted with 5 μL of freshly prepared CuAAC reaction cocktail. After 1 h at room temperature, samples were chloroform-methanol precipitated. Air-dried protein pellets were resuspended in 20 μL SDS-HEPES buffer and diluted to 0.25 mg/mL with HEPES buffer. Protein samples were then added to 10 μL of high-capacity NeutrAvidin beads, and incubated with end-over-end rotation for 90 min at room temperature. The beads were sequentially washed with 1 mL of 0.4% SDS in PBS (2 × 5 min), 4 M urea in PBS (2 × 5 min) and PBS (4 × 3 min).

For SDS-PAGE and Western blot analyses, 25–50 μL of freshly prepared elution buffer (25 mM Na_2_S_2_O_4_ in PBS with 0.1% SDS) were added to the beads and the samples were eluted by 1 h incubation at room temperature. For LC-ESI MS/MS analysis, samples were reduced with 80 μL of 10 mM TCEP (pH 8 in AMBIC) for 30 min at room temperature. After removing the supernatant and washing the beads once with AMBIC, samples were incubated with 80 μL 10 mM iodoacetamide in AMBIC (ThermoFisher Scientific, A39271) at room temperature in the dark for 30 min. The supernatant was removed and the beads were washed thrice with AMBIC. Finally, 80 μL of AMBIC containing trypsin (Promega, V5280) was added for on-bead digestion overnight at 37 °C. Supernatants were transferred into clean low binding tubes (Eppendorf, 022431081). The beads were washed with 80 μL of 1% formic acid (FA)/15% acetonitrile (ACN) in H_2_O, followed by 80 μL of 1% FA in H_2_O. These washes were combined with the supernatants and the peptides were concentrated and desalted using C18 spin tips (ThermoFisher Scientific, 84850). Peptides were dried in the speedvac and resuspended prior to LC-ESI MS/MS analysis.

### LC-ESI-MS/MS analysis by Orbitrap Elite MS

Three independent proteomics runs were performed on the same Orbitrap Elite hybrid mass spectrometer (Thermo Electron, Bremen, Germany) equipped with a PicoView nanospray interface (New Objective, Woburn, MA) connected to following LC systems. For the first and second runs, NanoLC−nanoESI-MS/MS analysis was performed on a nanoAcquity system (Waters, Milford, MA) connected to the Orbitrap mass spectrometer. Peptide mixtures were loaded onto a 75 μm ID, 25 cm length C18 BEH column (Waters, Milford, MA) packed with 1.7 μm particles with a pore with of 130 Å. In the first run, peptides were separated using a segmented gradient in 30 min from 5 to 35% solvent B (ACN with 0.1% FA) at a flow rate of 300 nL/min and a column temperature of 35 °C. Solvent A was 0.1% FA in water. For the second run, peptides were separated using a segmented gradient from 5 to 25% (0 to 82.5 min), 25 to 35% (82.5 to 90 min) solvent B (ACN with 0.1% FA) at a flow rate of 300 nL/min and a column temperature of 35 °C. Solvent A was 0.1% FA in water. For the third run, NanoLC−nanoESI-MS/MS analysis was performed on an EASY-nLC™ 1000 system (Waters, Milford, MA) connected to the Orbitrap mass spectrometer. Peptide mixtures were loaded onto a 75 μm ID, 25 cm length PepMap C18 column (ThermoFisher Scientific) packed with 2 μm particles with a pore with of 100 Å and were separated using a segmented gradient from 5 to 32% (0 to 82.5 min), 32 to 45% (82.5 to 90 min) solvent B (80% ACN with 0.1% FA) at a flow rate of 300 nL/min and a column temperature of 35 °C. Solvent A was 0.1% FA in water. For all three proteomics runs, the mass spectrometer was operated in the data-dependent mode. Briefly, survey full scan MS spectra were acquired in the Orbitrap (*m*/*z* 350–1600) with the resolution set to 120 K at *m*/*z* 400 and automatic gain control (AGC) target at 10^6^. The 20 most intense ions were sequentially isolated for CID MS/MS fragmentation and detection in the linear ion trap (AGC target at 10^4^) with previously selected ions dynamically excluded for 60 s. Ions with singly and unrecognized charge state were also excluded.

### MS data processing and database search for protein identification

MS and MS/MS raw data were processed by Proteome Discoverer (v2.5.0; Thermo Scientific, Waltham, MA, USA) and searched against Swiss-Prot protein sequence database and cRAP contaminant database with the Mascot server (v.2.8.0; Matrix Science, Boston, MA, USA). Taxonomy was set as Mus. Search criteria used were trypsin digestion, static modifications set as carbamidomethyl (C), dynamic modifications set as oxidation (M) and allowing up to 2 missed cleavage, mass accuracy of 10 ppm for the parent ion and 0.6 Da for the fragment ions mass tolerance. The overall false discovery rate was set as 1%. Proteins with two peptides and at least one unique peptide were retained in this study.

### Bioinformatics analysis of candidate excB targets

Each proteomics run was an independent biological repeat. In each of the three proteomics runs, from the list of proteins obtained in the probe-labeled samples (10 µM excB-16*C*-AOyne; 10 µM excB-12*O*-PZyne), we excluded proteins that were also identified (>0.25-fold peptide spectral match (PSM) compared to probe-labeled samples) in either of the two negative control samples (10 µM excB; 50 µM excB) as well as samples that were pretreated with biologically active analogs prior to probe labeling (10 μM probe + pretreat 40 μM excB; 10 μM probe + pretreat 40 μM excB-16*C*-AO or excB-12*O*-PZ). The same filter criteria were applied to the list of proteins identified in samples pretreated with the biologically inactive analog BExcL prior to probe labeling (10 μM probe + pretreat 40 μM BExcL). For probes excB-16*C*-AOyne and excB-12*O*-PZyne, filtering for proteins that were identified ≥4 times in a total of 6 samples over three proteomics runs yielded 24 and 16 proteins, respectively. These proteins were represented in venn diagram of Fig. 3b and listed in Supplementary Data 2.

### Wild type and mutant STING expression constructs

The murine STING (mSTING) gene was PCR amplified from cDNA of RAW 264.7 cells and cloned into the *Hind*III/*Xba*I site of pCMV3 vector (SinoBiological) with and without a C-terminal HA tag. The human STING (hSTING, R232 isoform) was PCR amplified from the genomic DNA of a stable Jurkat cell line expressing hSTING-GFP, which was obtained from Dr. Ya-Hui Chi’s laboratory, and similarly cloned into the *Hind*III/*Xba*I site of pCMV3. Cysteine-to-serine STING mutants were generated using the QuikChange Lightning Multi Site-Directed Mutagenesis Kit (Stratagene, 210513). Primers used in this study can be found in Supplementary Data 3. All constructs were validated by Sanger sequencing. mSTING and hSTING constructs were transfected into HEK293T cells using the TransIT®-LT1 Transfection Reagent (Mirus Bio, MIR2300) for 16–24 h prior to competition and probe labeling experiments.

### STING immunoprecipitation for intact mass analysis

mSTING-HA or mSTING(C91S)-HA constructs were transfected into HEK293T cells for 24 h prior to treatment with 10 μM excB or DMSO. After 2 h incubation, cells were harvested, resuspended in lysis buffer (20 mM HEPES pH 7.4, 150 mM NaCl, 10% glycerol and 1% n-dodecyl-β-D-maltoside) and sonicated at 50% amplitude for 10 min with 10 s intervals and 10 s pauses (Qsonica Q700). For mSTING-HA immunopurification, 1 mg of protein lysate was added to 60 μL of prewashed monoclonal anti-HA magnetic beads (K0201, MedChemExpress) and incubated on a rotator for 2 h at 4 °C. The beads were subsequently washed trice with 25 mM AMBIC and four times with LC-MS grade water. Two sequential elutions were carried out, each time by incubating the beads with 50 μL of 0.5% FA/30% ACN and shaking at room temperature for 30 min. Eluents were pooled, dried in the speedvac and resuspended prior to intact ESI-MS analysis.

### Intact mass analysis of mSTING

A Thermo Q-Exactive Plus mass spectrometer coupled with C4 column (Waters; 2.1 mm × 50 mm; Part No. 186004495) in high mass range mode was used for LC-MS analysis. The column temperature was maintained at 60 °C with flow rate at 0.25 mL/min using Buffer A (0.1% FA) and Buffer B (0.1% FA in ACN). The 20 min run was performed through gradient 15 to 60% ACN in 9 min, 2 min increase to 90% ACN followed by washout for 2 min at 90% ACN, and re-equilibration at 15% ACN for 5 min. Spray voltage was set to 3.5 kV, S-lens RF level at 100, and heated capillary at 253 °C. In source CID was 10 eV. Full scan resolution was set to 17.5 K at *m*/*z* 200 with 10 average microscans. Target value was set at 3 × 10^6^ with a maximum injection time of 200 ms. Mass range was set to 500–5000 *m*/*z*. All data was acquired in profile mode using positive polarity. LC-MS results were deconvoluted using Intact Protein Analysis in BioPharma Finder 3.2 software. Data were manually confirmed.

### hSTING-ExcB protein-ligand docking

The three-dimensional structures of excB was generated using Python programming language and the open-source RDKit library^48^. The protein structure of full-length hSTING for docking experiments was obtained from the Protein Data Bank (PDB) with PDB ID of 6NT5^49^. The protein structure was prepared and protonated with AMBER-FB15 force field^50^. Protein-ligand blind docking was conducted to analyze potential binding pockets and protein-ligand interactions using a three-step method. The cavities of protein surface were firstly determined with CB-Dock^51^. Secondly, the AutoDock Vina was employed to dock excB following the protein-ligand docking protocol^52^. Finally, based on the previous blind docking result, a covalent docking on Cys91 residue was conducted with explicitly specified binding site flexibility for four arginines residues (Arg83, Arg86, Arg94, and Arg95) using the AutoDockFR with the AutoDock4 scoring function following the covalent docking protocol^53^. The covalent docking poses of the hSTING-Cys91-ExcB protein-ligand structure with lowest energy were obtained from the analysis. To further calculate the membrane embedding structure of the hSTING-Cys91-ExcB, the Bilayer Builder function in the CHARMM-GUI was performed^54^, where PPM 2.0 was utilized for protein-membrane orientation calculation^55^, and heterogeneous lipids with simplified composition of mammalian endoplasmic reticulum membranes^56^, were utilized to simulate the membrane structure. The protein-ligand binding pose analysis and figure generation were done using PyMOL v2.5.4.

### Detection of STING palmitoylation

HEK293T cells expressing wild type or mutant mSTING proteins were labeled with 50 μM of alk-16 (RL-2065, Iris Biotech GmbH) for 2 h. For experiments involving inhibitors, the cells were pretreated with 10–40 μM of the indicated inhibitors for 1.5 h prior to metabolic labeling with 50 μM alk-16 for 3.5 h. Cell harvesting, lysate preparation, CuAAC and monitoring of mSTING palmitoylation by Western blot or in-gel fluorescence were performed as described above.

### Analysis of STING signaling in NIH3T3 cells

NIH3T3 cells were seeded at a density of 7 × 10^5^ cells in 2 mL in 6-well plates for 18 h before treatment. Cells were pretreated with indicated concentrations of excB or equal volume of vehicle control (DMSO) for 1 h prior to stimulation with 4 µg/mL of dsDNA (pcDNA3.1) delivered to cells using Lipofectamine 2000 (Invitrogen). At different time points, cells were harvested and washed thrice with ice-cold PBS and pelleted at 400 × *g* for 5 min. Cells were flash-frozen in liquid nitrogen and stored at −80 °C prior to lysis. As described above, frozen cell pellets were lysed in SDS lysis buffer containing phosphatase inhibitors (Roche, 4906845001) and protein concentrations were determined using the BCA protein assay. Proteins were then separated by SDS-PAGE and transferred onto PVDF membranes (Millipore, IPVH85R) and probed for TBK1, phospho-TBK1, IRF3, phospho-IRF3 and α-tubulin. Blots were visualized using SuperSignal™ West Pico PLUS Chemiluminescent Substrate (ThermoFisher Scientific, 34580) and imaged on a ChemiDoc MP imaging system (Bio-Rad).

### Antibodies used for western blot analyses

The antibodies used in this study for Western blots were as follows. Anti-STING (#13647, 1:1000 dilution), anti-phospho-TBK1 (#5483, 1:1000 dilution), anti-TBK1 (#3504, 1:1000 dilution), anti-phospho-IRF3 S396 (#29047, 1:1000 dilution), anti-IRF3 (#4302, 1:1000 dilution), anti-α-tubulin (#2144, 1:2000 dilution) were purchased from Cell Signaling Technology. Anti-eIF2D (#12840-a-AP, 1:2000 dilution) was purchased from Proteintech. Anti-rabbit-HRP (#111-035-003, 1:20000 dilution) were purchased from Jackson ImmunoResearch Laboratories.

### IRF-Lucia reporter assay

THP1-Dual™ KI-hSTING-R232 NF-κB-SEAP and IRF-Lucia reporter monocytes (InvivoGen, thpd-r232) were seeded in flat-bottom 96-well plate at density of 5.0 × 10^5^/mL. Cells were treated with 5–40 µM excB for 2.5 h, followed by treatment with 100 µg/mL 2'3’-cyclic guanosine monophosphate–adenosine monophosphate (cGAMP, InvivoGen, tlrl-nacga23). All wells, including the DMSO vehicle control, contained 0.08% (v/v) DMSO. Cell supernatants were harvested after 24 h incubation at 37 °C in a CO_2_ incubator. Luciferase activities were determined using the QUANTI-Luc™ reagent (InvivoGen, rep-qlc) and measured on a Perkin Elmer Wallac Victor^2^ 1420 Multilabel Counter. Interferon regulatory transcription factor (IRF) reporter activity was normalized to DMSO-treated controls samples using the following calculation. %IRF activity = ((signal(drug) – (cell blank control))/(signal(DMSO) − (cell blank control)))*100%. Researchers performing the assay were blind to the identity and nature of the compounds tested (excB was one of many compounds assayed and was labeled with a generic number).

### Statistical analyses

iNOS gene expression, nitric oxide production and cell viability data for multiple different treatment groups were compared using one-way analysis of variance (ANOVA) followed by the Tukey post-hoc test using GraphPad Prism v8.4.2. *P*-values of <0.05 was considered statistically significant. Three independent biological repeats were performed for each experiment.

### Reporting summary

Further information on research design is available in the Nature Portfolio Reporting Summary linked to this article.

## Supplementary information

Supplementary Information

Description of Additional Supplementary Files

Supplementary Data 1

Supplementary Data 2

Supplementary Data 3

Supplementary Data 4

Reporting Summary

## Data Availability

The mass spectrometry proteomics data have been deposited to the ProteomeXchange Consortium via the PRIDE partner repository^57^, with the dataset identifier PXD042748. The molecular docking codes and data have been deposited on GitHub with Zenodo DOI: 10.5281/zenodo.7993805. NMR spectra can be found in Supplementary Data 1. List of candidate cellular targets of excB identified from RAW 264.7 macrophages is found in Supplementary Data 2. Primers used in this study are provided in Supplementary Data 3. Source data and specific data *P*-values are included in Supplementary Data 4.
