# Supplementary material for: Marine diterpenoid targets STING palmitoylation in mammalian cells
Source: Commun Chem. 2023 Jul 18;6:153. doi: 10.1038/s42004-023-00956-9 (PMC10354091; doi:10.1038/s42004-023-00956-9)
Supplement: Supplementary file 1 — Supplementary Information [file 42004_2023_956_MOESM1_ESM.pdf]

Supporting Information

Marine Diterpenoid Targets STING Palmitoylation in Mammalian Cells

Wan-Chi Hsiao[a,b#], Guang-Hao Niu[c#], Chen-Fu Lo[c], Jing-Ya Wang[c], Ya-Hui Chi[c], Wei-Cheng Huang[c], Chun-Wei Tung[c], Ping-Jyun Sung[d,e,f,g*], Lun Kelvin Tsou[c*], Mingzi M. Zhang[a*]

[a]Institute of Molecular and Genomic Medicine, National Health Research Institutes, Miaoli, 35053, Taiwan

[b]Institute of Biotechnology, National Tsing Hua University, Hsinchu, 30013, Taiwan

[c]Institute of Biotechnology and Pharmaceutical Research, National Health Research Institutes, Miaoli, 35053, Taiwan

[d]Department of Marine Biotechnology and Resources, National Sun Yat-sen University, Kaohsiung 804201, Taiwan

[e]National Museum of Marine Biology and Aquarium, Pingtung 944401, Taiwan

[f]Chinese Medicine Research and Development Center, China Medical University Hospital, Taichung 404394, Taiwan

[g]Graduate Institute of Natural Products, Kaohsiung Medical University, Kaohsiung 807378, Taiwan

[#]These authors contributed equally

*To whom correspondence may be addressed:

zhangmz@nhri.edu.tw, 35 Keyan Road, Miaoli, 35053, Taiwan

kelvintsou@nhri.edu.tw, 35 Keyan Road, Miaoli, 35053, Taiwan

pjsung@nmmba.gov.tw, National Museum of Marine Biology and Aquarium, Pingtung 944401, Taiwan

Supplementary Figures **Page S3-S14**

Supplementary Methods **Page S15-S23**

Supplementary References **Page S24**

Supplementary Figures

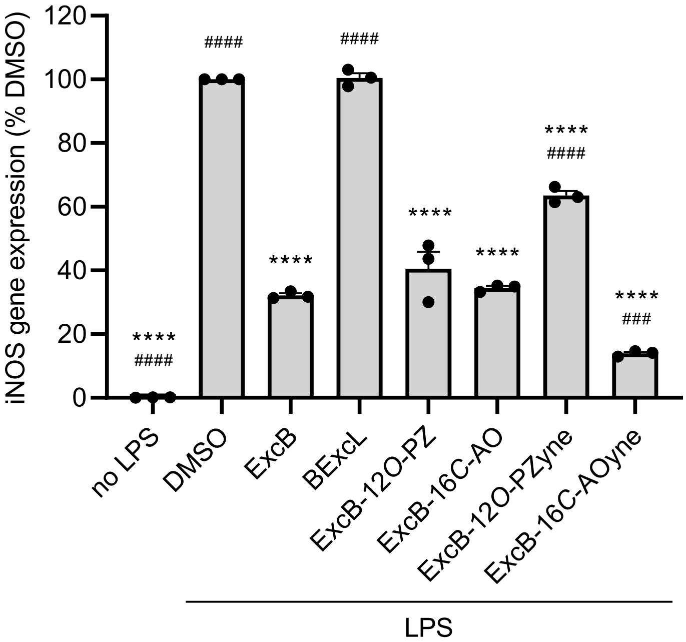

Supplementary Figure 1. Effect of excB analogs and probes on LPS-induced *iNOS* expression in RAW 264.7 macrophages. Cells were treated with LPS and 50 µM of the indicated compounds for 8 h. Relative *iNOS* expression compared to the DMSO control was determined by qRT-PCR (β-actin as internal control). n=3. Error bars, s.e.m. ###P<0.001, ####P<0.0001 compared to excB. ****P<0.0001 compared to DMSO.

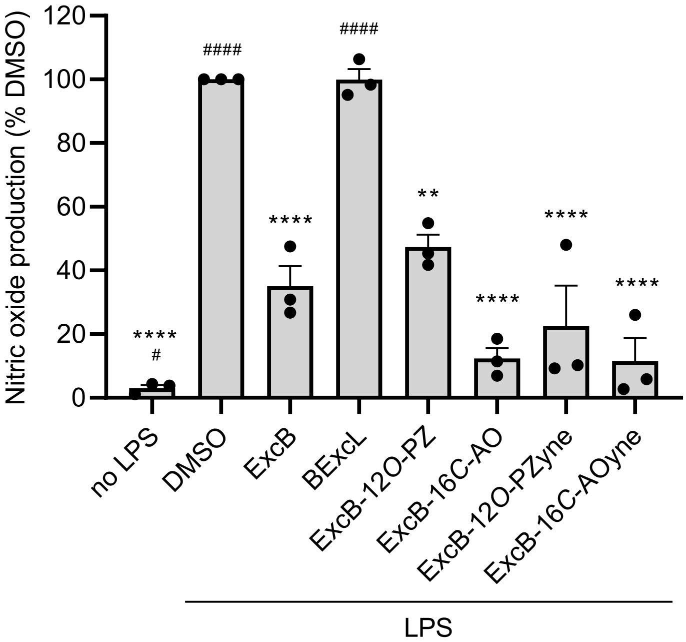

Supplementary Figure 2. Effect of excB analogs and probes on LPS-induced nitric oxide production in RAW 264.7 macrophages. Cells were treated with LPS and 10 µM of the indicated compounds for 24 h. Relative nitric oxide levels in culture supernatants compared to DMSO were determined using a modified Griess assay. n=3. Error bars, s.e.m. #P<0.05, ####P<0.0001 compared to excB. **P<0.01, ****P<0.0001 compared to DMSO.

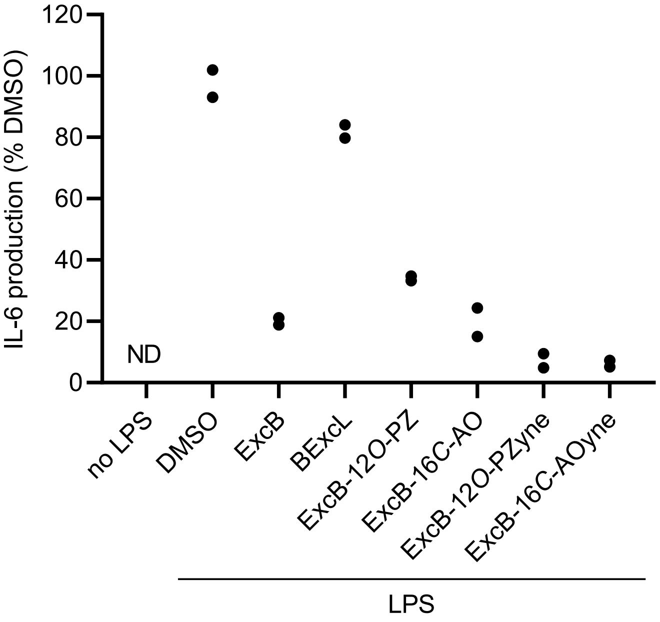

Supplementary Figure 3. Effect of excB analogs and probes on LPS-induced IL-6 production in RAW 264.7 macrophages. Cells were treated with LPS and 10 µM of the indicated compounds for 24 h. Relative IL-6 levels in culture supernatants compared to DMSO were determined using ELISA. n=2. ND, not detected.

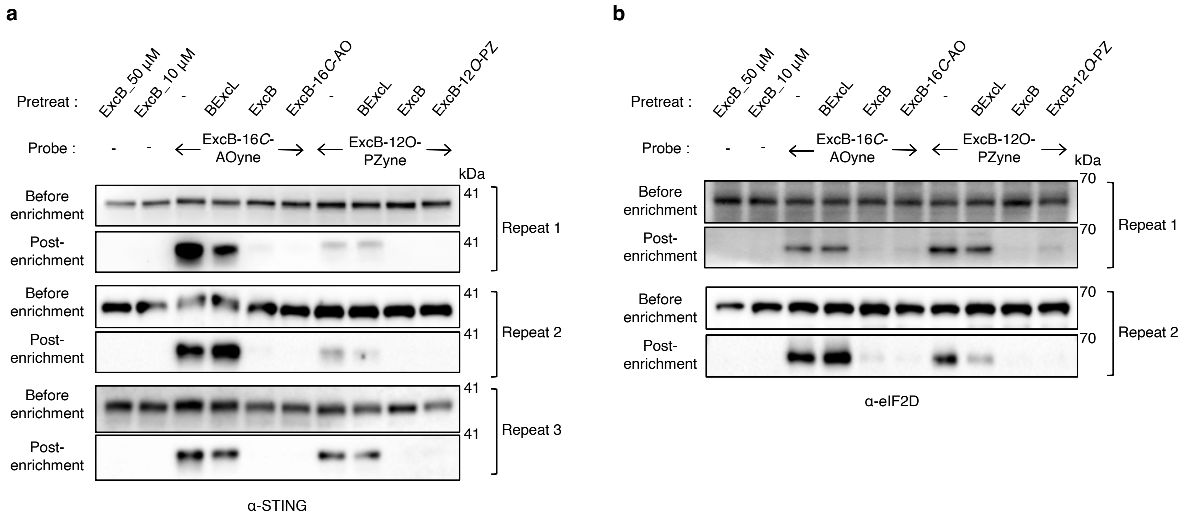

Supplementary Figure 4. Western blot validation of STING and eIF2D as excB targets in RAW 264.7 macrophages. Cells were pretreated for 2 h with 40 µM of excB or indicated analogs prior to 1 h labelling with 10 µM excB-16*C*-AOyne or excB-12*O*-PZyne. Cell lysates were reacted with azide-biotin and samples before and after NeutrAvidin enrichment were immunoblotted for (**a**) STING or (**b**) eIF2D. Each repeat is an independent biological repeat. These data are independent from data in Figure 3c.

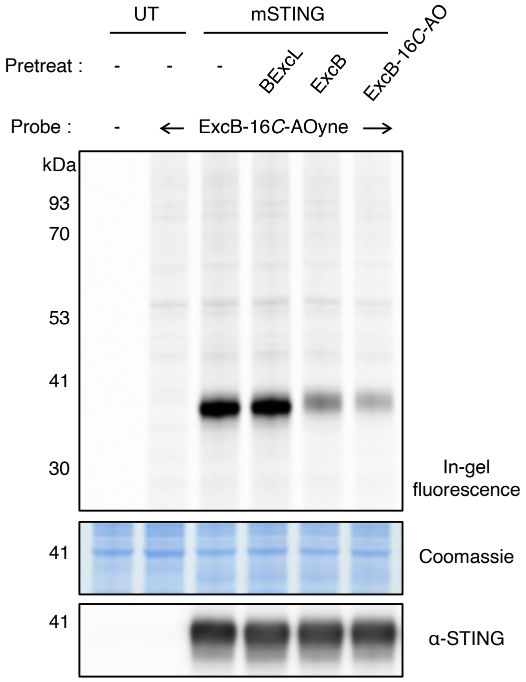

Supplementary Figure 5. Fluorescent detection of excB-16*C*-AOyne engagement of mSTING in HEK293T cells. Cells expressing mSTING were pretreated with 40 µM of the indicated excB analogs prior to labeling with 10 µM excB-16*C*-AOyne for 1 h. Probe engagement of mSTING was monitored by in-gel fluorescence. Coomassie stain and anti-STING blot act as loading controls for the accompanying fluorescence gel with selected protein molecular weight markers. UT, untransfected control. Western blot and in-gel fluorescence data were representative of 2 independent biological repeats.

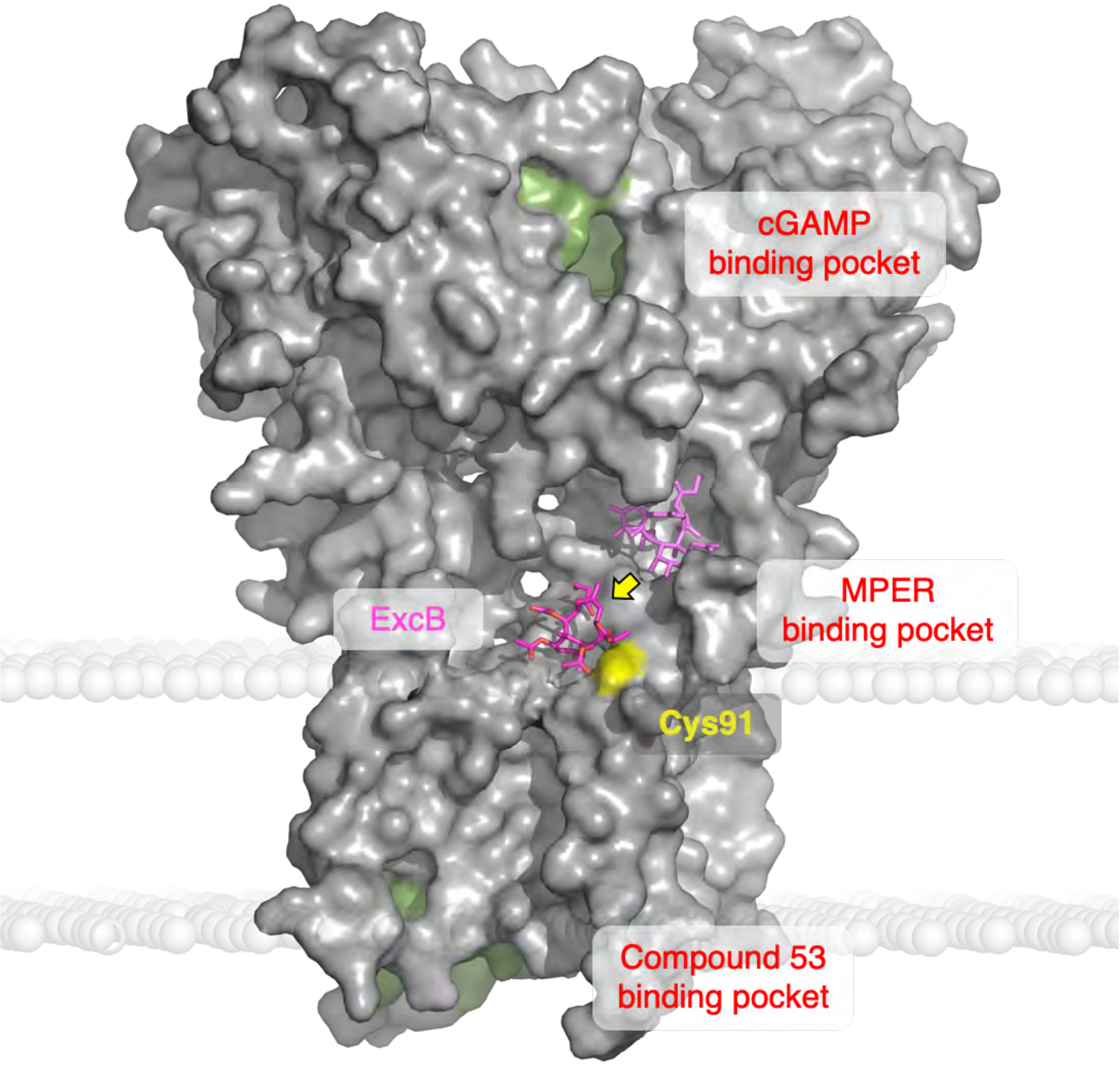

Supplementary Figure 6. Molecular docking revealed three potential excB binding pockets on full length apo-hSTING (6NT5). The structure of excB (pink) is only shown for the MPER binding pocket close to Cys91 (yellow). Positioning of the membrane bilayer (light gray) was calculated using PPM2.0. Covalent docking of excB (magenta) at Cys91 was superimposed in this structure and indicated by the yellow arrow. The other two potential binding pockets were indicated in green. MPER, membrane-proximal external region.

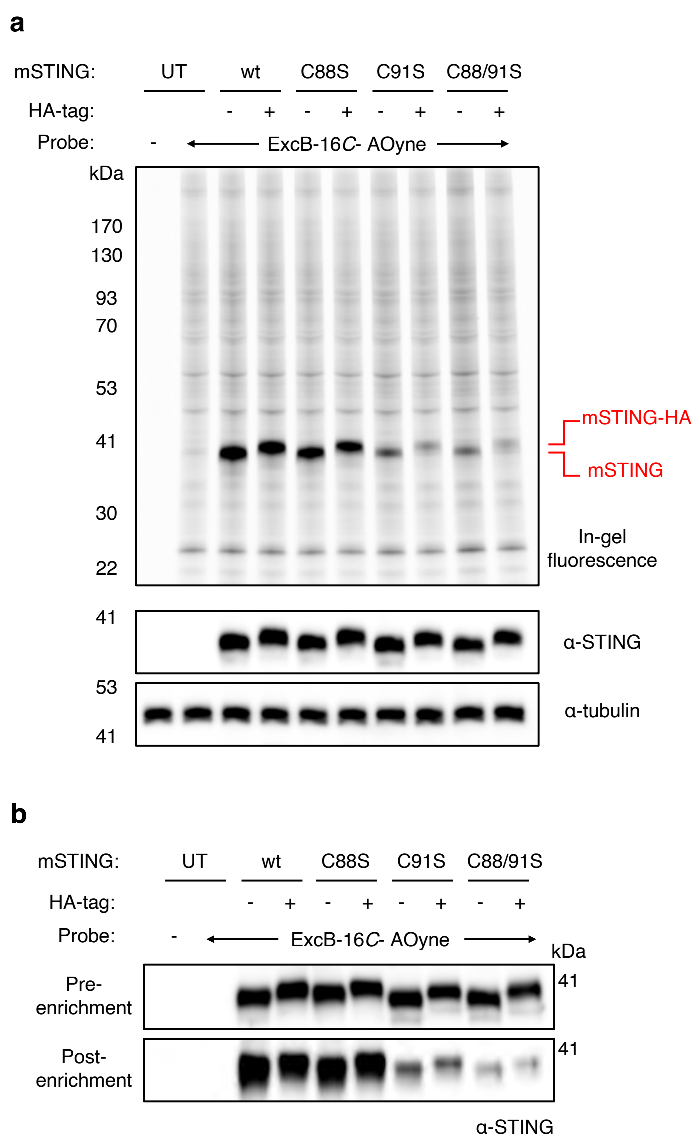

Supplementary Figure 7. Cys91 is important for excB-16*C*-AOyne engagement of mSTING. (**a**) Probe engagement of mSTING and mSTING-HA (indicated by red lines) in HEK293T cells was monitored by in-gel fluorescence. Anti-STING and anti-tubulin blots act as loading controls for fluorescence gel. Selected protein molecular weight markers are indicated. (**b**) Probe engagement of mSTING and mSTING-HA was monitored by Western blot before and after NeutrAvidin enrichment following reaction of lysates with azide-biotin. UT, untransfected.

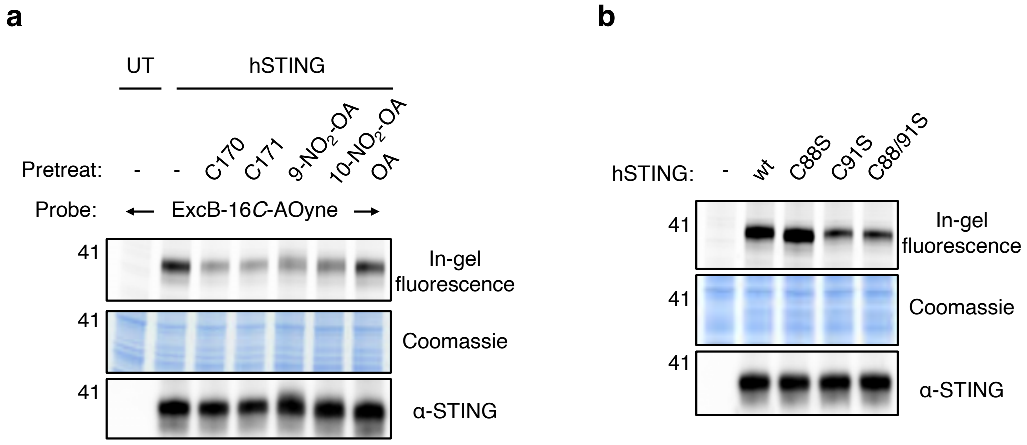

Supplementary Figure 8. ExcB covalently engages human STING (hSTING). (**a**) HEK293T cells expressing hSTING were pretreated for 2 h with 10 µM of the indicated compounds prior to 15 min labelling with 10 µM excB-16*C*-AOyne. Probe labeling of hSTING was monitored by in-gel fluorescence. UT, untransfected. (**b**) HEK293T cells expressing wild type (wt) hSTING and indicated cysteine-to-serine mutants were labeled with 10 µM excB-16*C*-AOyne for 1 h. Probe labeling of hSTING was monitored by in-gel fluorescence. Anti-STING and Coomassie stain act as loading controls. Selected protein molecular weight markers are indicated. Western blot data were representative of two independent biological repeats.

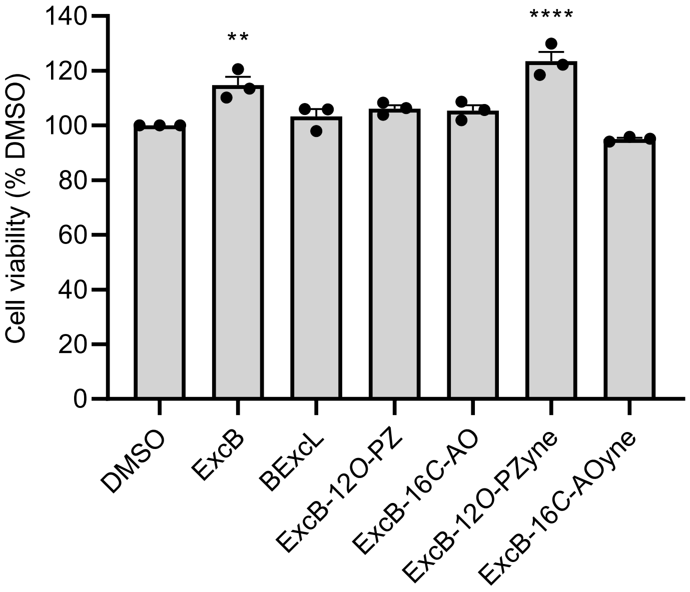

Supplementary Figure 9. Effect of excB and indicated analogs and probes on the viability of PMA-induced THP-1 macrophages. Cells were treated with 10 µM of indicated compounds for 24 h. Cell viability compared to DMSO control was determined using the MTS assay. n=3. Error bars, s.e.m. #P<0.05, **P<0.01, ****P<0.0001 compared to DMSO.

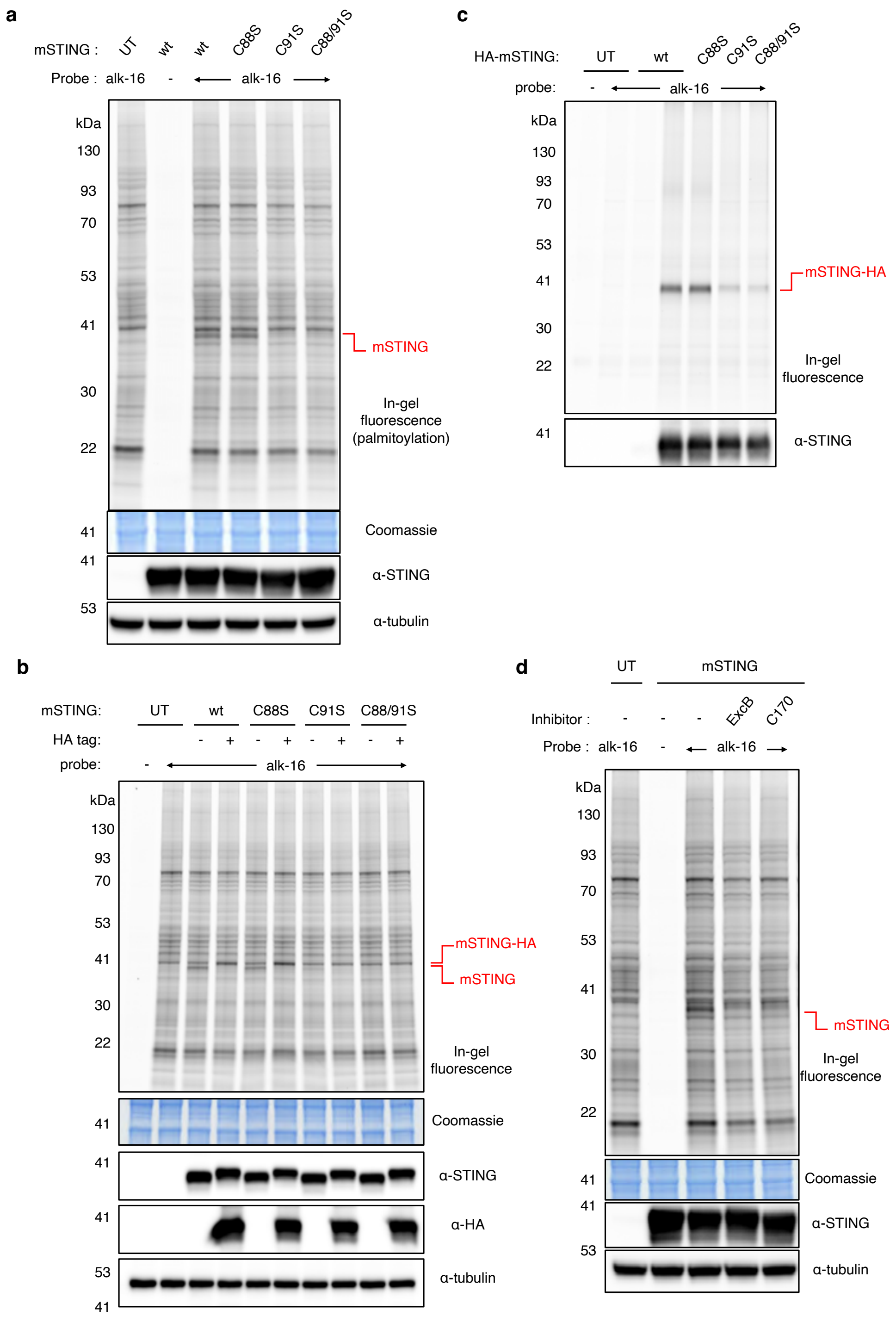

Supplementary Figure 10. Fluorescent detection of mSTING palmitoylation using alk-16. (**a, b**) HEK293T cells expressing wild type (wt) mSTING or indicated cysteine mutants with or without HA tags were labelled with 50 µM alk-16 for 2 h. mSTING and mSTING-HA palmitoylation (indicated by red lines) was monitored by in-gel fluorescence. (**c**) The indicated mSTING-HA proteins were immunopurified from alk-16 labelled HEK293T cell lysates prior to reaction with azide-Cy5 and in-gel fluorescence detection. (**d**) HEK293T cells expressing mSTING were pretreated for 1.5 h with 40 µM of indicated inhibitors prior to metabolic labeling with 50 µM alk-16 for 3.5 h. Coomassie staining, anti-tubulin, anti-HA and anti-STING blots act as loading controls for accompanying fluorescence gels with selected protein molecular weight markers. UT, untransfected.

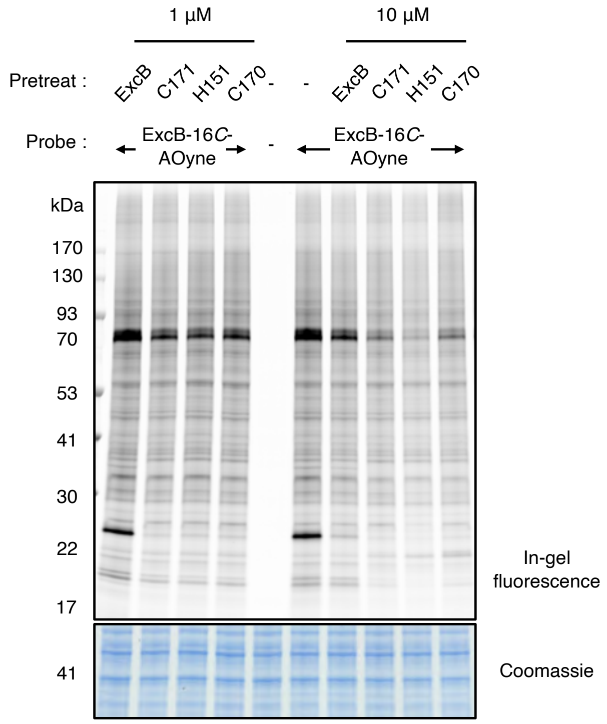

Supplementary Figure 11. Competition of excB-16*C*-AOyne labeling by STING inhibitors. RAW 264.7 macrophages were pretreated with 1 or 10 µM excB or indicated STING inhibitors for 2 h prior to labeling with 10 µM excB-16*C*-AOyne for 15 min. Probe labeling of endogenous proteins was monitored by in-gel fluorescence. Coomassie stain act as loading controls. Selected protein molecular weight markers are indicated.

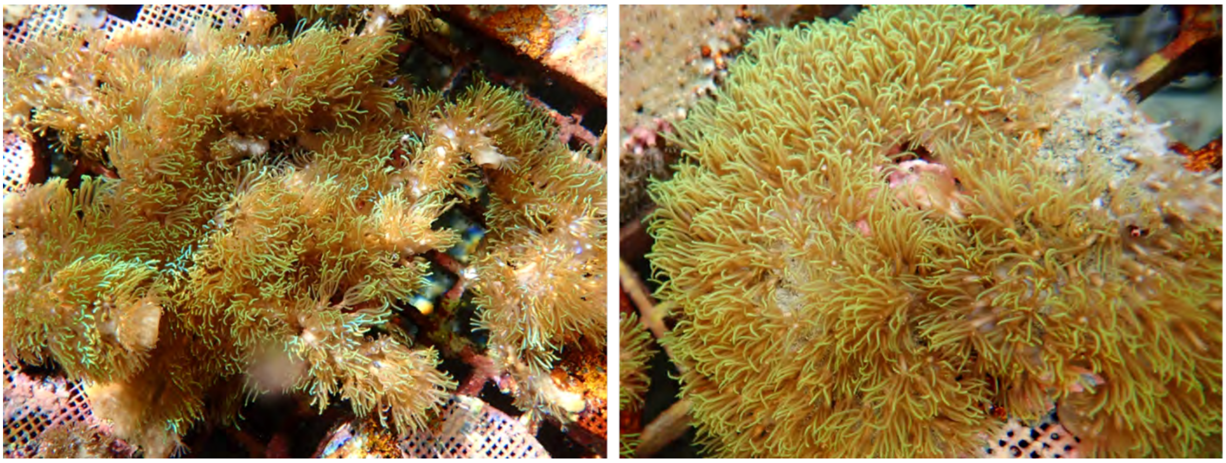

Supplementary Figure 12. Mariculture of *Briareum stechei.*

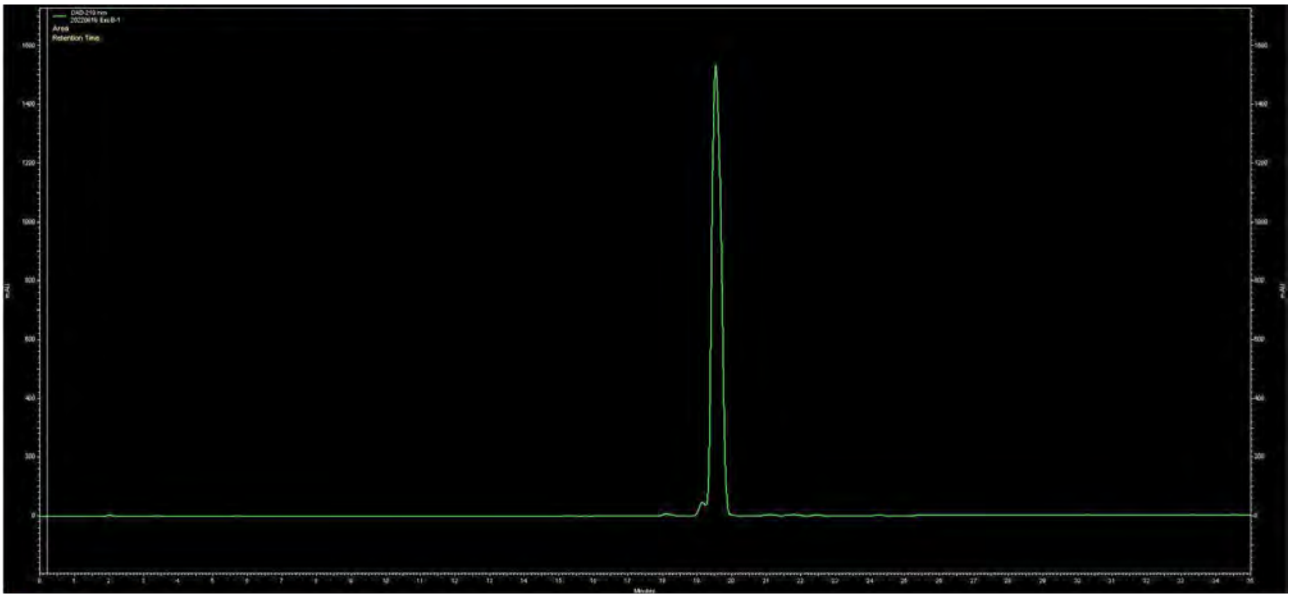

Mobile phase: 30% ACN_{aq} 0–5 min, 30%–100% ACN_{aq} 5–25 min, 100% ACN 25–35 min; 1 mL/min.

Supplementary Figure 13. HPLC analysis of isolated excB. ExcB was isolated from *B. stechei* with >95% purity based on HPLC analysis with detection at 210 nm. See Materials and Methods for details of excB isolation and analysis.

Supplementary Methods

Monitoring *iNOS* expression by qPCR

RAW 264.7 cells were seeded 18 h before treatment in a 6-well plate at a density of 7 × 10^5 cells in 2 mL per well. Cells were pretreated with 50 μM of the indicated compounds or vehicle control (DMSO) for 15 min prior to LPS (Sigma-Aldrich, L2880) treatment at 10 ng/mL. After 8 h, total RNA was extracted using RNeasy mini kit (Qiagen, 74106) with an additional on-column DNase (Qiagen) treatment step. Complementary DNA (cDNA) synthesis was carried out with 1 μg of total RNA using the RevertAid First Strand cDNA Synthesis Kit (ThermoFisher Scientific, K1622) following manufacturer's instructions. Quantitative real-time polymerase chain reaction (qPCR) assays were performed in triplicates using QuantiNova SYBR Green PCR Kit (Qiagen, 208052) in 384-well plates (Axygen, PCR-384M2-C) at a final reaction volume of 10 μL per well (5 μL of SYBR Green Master Mix, 0.05 μL of QN ROX dye, 0.7 μL each of 10 μM forward and reverse primer stock solutions, 1 μL of 10-fold diluted cDNA, and 2.55 μL of RNase-free water). qPCR primers used in this study can be found in Supplementary Data 3. Reactions were monitored on the ViiA®7 Real-Time PCR Systems (Applied Biosystems) using the following cycling conditions: 95 °C for 3 min; 40 cycles of 95 °C for 10 s → 60 °C for 30 s; a melt curve stage of 95 °C for 15 s → 60 °C for 60 s, followed by a final dissociation step of 0.05 °C/s up to 95 °C and maintained for 15 s. Results were analyzed with the ViiA™ 7 software (Applied Biosystems). Relative *iNOS* expression was calculated using the comparative CT ($2^{-\Delta\Delta Ct}$) method with β-actin as the internal control.[1] Results were shown as % *iNOS* expression compared to DMSO-treated samples.

Nitric oxide production assay

Nitric oxide production was quantified using a modified Griess assay for nitrite quantification.[2] Briefly, 50 μL of 40 mg/mL Griess reagent (Sigma-Aldrich, G4410) was added to 50 μL of culture supernatants in clear 96-well plates. A standard curve of sodium nitrite (Sigma-Aldrich, 237213) covering the concentration range of 50–0.78 μM was obtained by 2-fold serial dilution with culture medium only as blank. After 15 min incubation in the dark at room temperature, absorbance at 540 nm was measured on the TECAN Infinite 200 pro spectrophotometer. Absorbance values of samples were subtracted from the blank and then transformed in $NaNO_2$ concentration according to the standard curve linear regression fit. Results were shown as % $NaNO_2$ concentration compared to DMSO control (100%).

IL-6 ELISA

IL-6 was measured in the culture supernatants of LPS-treated RAW 264.7 cells with and without compound treatment. RAW 264.7 cells were seeded in a 12-well plate at a density of 4 × 10^5 cells in 1 mL per well overnight. These cells were grown in serum-free DMEM for 2 h prior to compound or DMSO treatment at 10 μM for 1 h, after which the cells were treated with 10 ng/mL of LPS at a final volume of 0.6 mL per well. After 24 h incubation, culture supernatants were removed, centrifuged at 13, 000 g for 5 min and stored at -80 °C. Measurement of IL-6 production was performed using the Mouse IL-6 DuoSet ELISA kit (R&D Systems, DY406) according to manufacturer's instructions. Sample IL-6 concentrations were calculated using linear regression curve fit by plotting the log of IL-6 concentrations versus the log of the absorbance (450 nm) values. Results were shown as % IL-6 concentration compared to DMSO-treated samples.

STING immunoprecipitation for click chemistry reaction

HEK293T cells expressing mSTING-HA were harvested and lysed in RIPA buffer (25 mM Tris-HCl, pH 7.6, 150 mM NaCl, 1% NP-40, 1% sodium deoxycholate, 0.1% SDS). Cell debris was removed by centrifugation at 13,000 g for 5 min. For immunoprecipitation of mSTING-HA, 50 μg cell lysate was added to 10 μL of prewashed anti-HA magnetic beads (K0201, MedChemExpress) and incubated with end-over-end rotation for 2 h at 4 °C. The beads were subsequently washed twice with PBST (0.5% Tween-20 in PBS, pH 7.4) and thrice with PBS. For in-gel fluorescence detection experiments, the beads were resuspended in 20 μL PBS and reacted with 2.475 μL of freshly prepared CuAAC reaction cocktail for 1 h at room temperature. After washing twice with PBS, the beads were resuspended in 20 μL PBS, 6.96 μL 4× SDS-loading buffer and 1.04 μL TCEP. Samples were heated at 95 °C for 5 min prior to SDS-PAGE separation.

MTS cellular viability assay

THP-1 cells were cultured in Roswell Park Memorial Institute (RPMI, Hyclone) 1640 supplemented with 10% FBS, penicillin (100 U/mL), streptomycin (100 μg/mL). THP-1 monocytes were seeded into 96-well plates (5 x 10^4 cells/well) and differentiated into adherent macrophages by 48 h incubation with 20 nM phorbol 12-myristate 13-acetate (PMA). PMA-induced THP-1 cells about 80% confluence were treated with 10 μM of indicated compounds or vehicle (DMSO) prepared in culture medium to a final volume of 100 μL for 24 h. Cell viability was analyzed using the colorimetric CellTiter 96® AQueous MTS assay (Promega, G1111). For this, phenazine methosulfate (PMS, P9625) was made up in DPBS (0.92 mg/mL), sterile filtered and stored at –20 °C before use. A working solution was freshly prepared by mixing phenol red-free RPMI medium (Hyclone), MTS solution, and PMS solution in a ratio of 8:2:0.1. After removing culture supernatants, 120 μL of working solution was added to the cells. After 20–60 min incubation at 37 °C, absorbance was measured at 490 nm on the TECAN Infinite 200 Pro spectrophotometer. Results were shown as % viability compared to DMSO-treated cells. Each condition was carried out in triplicate.

Chemical synthesis of excB analogs and probes

Reactions were carried out under ambient atmosphere unless otherwise specified. Anhydrous dimethylformaldehyde (DMF) was purchased form Acros. Dry dichloromethane (CH_2Cl_2) was obtained by passing through activated aluminum column. All commercially obtained reagents were used as received unless otherwise specified. Yields refer to purified and spectroscopically pure compounds. All reactions were monitored by thin-layer chromatography (TLC) using Merck silica gel 60 F_{254} pre-coated glass plates and visualized by a combination of UV light (254 nm) and $KMnO_4$ staining. Flash column chromatography was performed using silica gel (SiliaFlash®P60, 40-63 μm) purchased from Silicycle. Eluent systems are given in volume/volume concentrations. NMR spectra were recorded on a Bruker Ascend™ 400 spectrometer operating at 400 MHz for ^{1}H and 100 MHz for ^{13}C, Bruker Ascend™ 600 operating at 600 MHz for ^{1}H and 151 MHz for ^{13}C acquisitions, respectively. Chemical shifts are reported in ppm with the solvent resonance as the internal standard. The following solvent chemical shifts were used as reference values (ppm): $CDCl_3$ = 7.26 (^{1}H), 77.0 (^{13}C); acetone-*d*$_6$ = 2.05 (^{1}H), 29.8 (^{13}C). Data is reported as follows: s = singlet, br = broad, d = doublet, t = triplet, q = quartet, m = multiplet; ABq = AB quartet; coupling constants in Hz; integration. All NMR spectra are found in Supplementary Data 1. Electrospray mass spectra (ESMS) were recorded as m/z values using an Agilent 1290 infinity II mass spectrometer. High-resolution mass spectra (HRMS) were obtained on JEOL JMS-700 (FAB) or Waters LCT (ESI) at the Academia Sinica.

ExcB

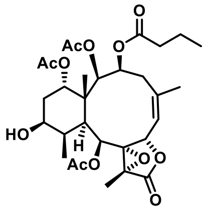

excB

^{1}H NMR (600 MHz, acetone-*d*$_6$, -40 °C) δ 5.85 – 5.80 (m, 1H), 5.67 (d, *J* = 7.4 Hz, 1H), 5.54 (d, *J* = 10.4 Hz, 1H), 5.35 (dt, *J* = 7.4, 1.8 Hz, 1H), 5.15 (d, *J* = 2.4 Hz, 1H), 4.67 (dd, *J* = 3.9, 2.2 Hz, 1H), 4.19 (d, *J* = 3.7 Hz, 1H), 4.01 (dd, *J* = 15.8, 7.5 Hz, 1H), 3.95 – 3.87 (m, 1H), 3.07 (dd, *J* = 10.5, 5.1 Hz, 1H), 2.54 – 2.45 (m, 1H), 2.39 (s, 3H), 2.22 (s, 3H), 2.20 (s, 3H), 2.19 – 2.15 (m, 1H), 2.14 – 2.06 (m, 2H), 1.95 (s, 3H), 1.88 (ddd, *J* = 14.3, 12.3, 2.3 Hz, 1H), 1.75 – 1.69 (m, 1H), 1.54 – 1.45 (m, 5H), 1.02 (d, *J* = 7.1 Hz, 3H), 0.85 (t, *J* = 7.4 Hz, 3H), 0.82 (s, 3H).

^{13}C{^{1}H } NMR (151 MHz, acetone-*d*$_6$, -40 °C) δ 172.4 (C), 172.1 (C), 171.9 (C), 170.7 (C), 170.1 (C), 140.0 (C), 122.6 (CH), 82.0 (CH), 81.6 (CH), 74.3 (CH), 73.7 (CH), 69.3 (C), 66.0 (CH), 65.7 (CH), 60.4 (C), 44.1 (C), 40.3 (CH), 36.0 (CH_2), 35.8 (CH), 34.4 (CH_2), 30.2 (CH_2), 22.7 (CH_3), 22.2 (2xCH_3), 21.4 (CH_3), 18.4 (CH_2), 18.3 (CH_3), 13.7 (CH_3), 10.0 (CH_3), 9.3 (CH_3).

Synthesis of **excB-12*O*-PZ**

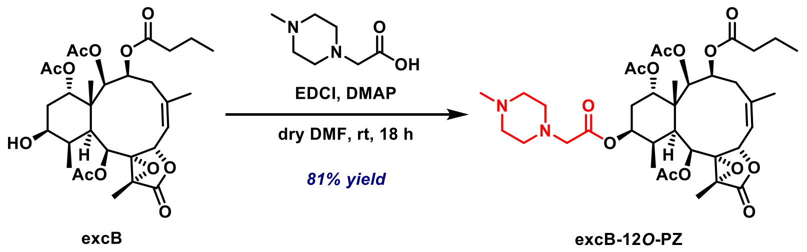

Under an ambient atmosphere, a 4 mL vial equipped with a stir bar was charged with **excB** (25.7 mg, 0.0432 mmol, 1.0 equiv.), 2-(4-methylpiperazin-1-yl)acetic acid (13.7 mg, 0.0864 mmol, 2.0 equiv.), DMAP (1.6 mg, 0.013 mmol, 0.3 equiv.), EDCI (24.8 mg, 0.130 mmol, 3.0 equiv.) and dried DMF (0.5 mL). The vial was sealed with a Teflon cap, and the resulting solution was stirred at room temperature. After 18 h, the reaction mixture was diluted with EtOAc and the organic layer was washed with H_2O. The organic layer was dried over $MgSO_4$, filtered and concentrated by rotary evaporation. The residue was purified by flash column chromatography (EtOAc : *n*-Hexane = 0 : 1→1 : 1→MeOH : CH_2Cl_2 = 1 : 10) to afford **excB-12*O*-PZ** (25.6 mg, 0.0348 mmol, 81% yield) as a yellowish oil.

TLC (MeOH : CH_2Cl_2 = 1 : 10): R_f = 0.50 ($KMnO_4$).
^{1}H NMR (600 MHz, acetone-d_6, -40 °C) δ 5.85 (d, *J* = 7.6 Hz, 1H), 5.67 (d, *J* = 7.3 Hz, 1H), 5.52 (d, *J* = 10.4 Hz, 1H), 5.36 (dd, *J* = 7.3, 2.0 Hz, 1H), 5.16 (d, *J* = 2.3 Hz, 1H), 5.07 – 4.98 (m, 1H), 4.70 (br s, 1H), 4.01 (dd, *J* = 15.8, 7.3 Hz, 1H), 3.21, 3.18 (ABq, *J* = 16.9 Hz, 2H), 3.17 (dd, *J* = 10.6, 5.2 Hz, 1H), 2.77 – 2.66 (m, 2H), 2.68 – 2.58 (m, 3H), 2.41 (s, 3H), 2.30 – 2.15 (m, 3H), 2.23 (s, 6H), 2.14 – 2.01 (m, 3H), 2.12 (s, 3H), 1.99 – 1.91 (m, 2H) 1.95 (s, 3H), 1.91 – 1.84 (m, 1H), 1.53 – 1.47 (m, 2H), 1.50 (s, 3H), 1.09 (d, *J* = 7.1 Hz, 3H), 0.86 (s, 3H), 0.85 (t, *J* = 7.3 Hz, 3H).
^{13}C{^{1}H } NMR (151 MHz, acetone-d_6, -40 °C) δ 172.2 (C), 172.1 (C), 171.9 (C), 170.7 (C), 170.1 (C), 169.9 (C), 140.0 (C), 122.6 (CH), 81.4 (2xCH), 74.2 (CH), 73.6 (CH), 70.2 (CH), 69.2 (C), 65.2 (CH), 60.6 (C), 59.0 (CH_2), 55.4 (CH_2), 52.8 (CH_2), 52.8 (CH_2), 46.2 (CH_3), 44.2 (C), 40.3 (CH), 35.9 (CH_2), 34.3 (CH_2), 33.0 (CH), 27.2 (CH_2), 22.6 (CH_3), 22.2 (2xCH_3), 21.4 (CH_3), 18.4 (CH_2), 18.0 (CH_3), 13.7 (CH_3), 10.3 (CH_3), 10.0 (CH_3).
HRMS-FAB (m/z) calcd for $C_{37}H_{55}N_2O_{13}Na$ $[M+Na]^+$ 735.3699, found 735.3694.

Preparation of **S4**[3]:

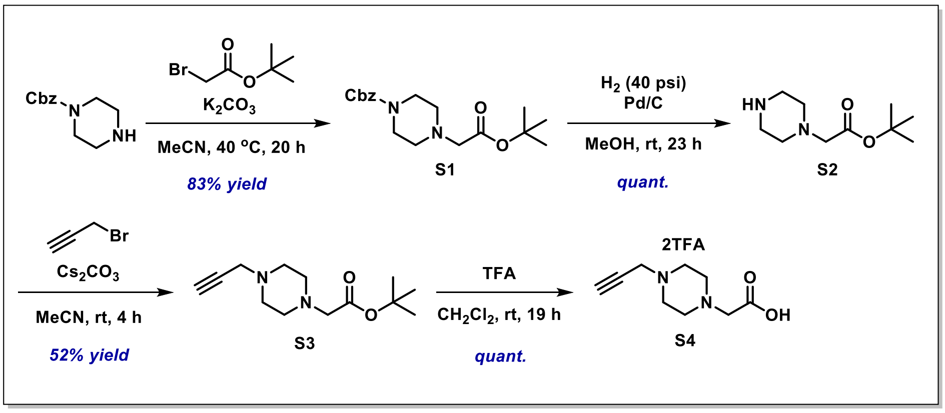

S1: To a solution of benzyl piperazine-1-carboxylate (2.00 g, 9.08 mmol, 1.0 equiv.) in MeCN (30 mL) was added K_2CO_3 (2.51 g, 18.16 mmol, 2.0 equiv.) and *tert*-butyl 2-bromoacetate (1.5 mL, d=1.32 g/mL, 9.99 mmol, 1.1 equiv.) at room temperature. The resulting mixture was stirred at 40 °C under N_2. After 20 h, the reaction mixture was cooled to room temperature, then filtered through a short pad of Celite. The filtrate was collected and concentrated by rotary evaporation. The residue was purified by flash column chromatography (EtOAc：*n*-Hexane = 0：1→1：5→1：3→1：1) to afford **S1** (2.53 g, 7.57 mmol, 83% yield) as a pale yellow oil.

TLC (EtOAc : *n*-Hexane = 1：1): R_f = 0.48 (UV_{254}, $KMnO_4$).
^{1}H NMR (400 MHz, $CDCl_3$, 22 °C) δ 7.42-7.28 (m, 5H), 5.12 (s, 2H), 3.61-3.49 (m, 4H), 3.12 (s, 2H), 2.54 (br s, 4H), 1.46 (s, 9H).

S2: **S1** (2.50 g, 7.48 mmol, 1.0 equiv.), MeOH (26 mL) and 10 wt% Pd/C (0.50 g) were added to a high-pressure vessel. The suspension was subjected to purge-charge cycle (3 times) with H_2 (20 psi). The reaction mixture was then shaken at room temperature under H_2 (40 psi) in the Parr hydrogenation apparatus. After 23 h, the reaction mixture was filtered through a pad of Celite, and the filtrate was collected and concentrated by rotary evaporation to afford a yellow oil **S2** (1.50 g, 7.48 mmol, quantitative yield). The crude product was directly used to the next step without further purification.

TLC (MeOH : CH_2Cl_2 = 1：10): R_f = 0.10 (UV_{254}, $KMnO_4$).
MS-ESI (m/z) calcd for $C_{10}H_{21}N_2O_2$ $[M+H]^+$ 201.2, found 201.3.

S3: To a solution of **S2** (0.820 g, 4.09 mmol, 1.0 equiv.) in MeCN (27 mL) was added Cs_2CO_3 (1.40 g, 4.29 mmol, 1.05 equiv.) and propargyl bromide (0.64 mL, d=1.335 g/mL, 4.29 mmol, 1.05 equiv.) at room temperature. The resulting mixture was stirred at room temperature under N_2. After 4 h, the reaction mixture was filtered through a short pad of Celite. The filtrate was collected and concentrated by rotary evaporation. The residue was purified by flash column chromatography (EtOAc：*n*-Hexane = 0：1→1：3→1：1(with 2% Et_3N)) to afford **S3** (0.507 g, 2.13 mmol, 52% yield) as a yellow solid.

TLC (EtOAc : *n*-Hexane = 1：1): R_f = 0.23 ($KMnO_4$).
^{1}H NMR (400 MHz, $CDCl_3$, 22 °C) δ 3.29 (d, *J* = 2.5 Hz, 2H), 3.11 (s, 2H), 2.65 (br s, 8H), 2.24 (t, *J* = 2.4 Hz, 1H), 1.46 (s, 9H).
^{1}H NMR (400 MHz, acetone-d_6, 22 °C) δ 3.26 (d, *J* = 2.3 Hz, 2H), 3.07 (d, *J* = 1.2 Hz, 2H), 2.68 (t, *J* = 2.4 Hz, 1H), 2.58 (br s, 4H), 2.54 – 2.48 (m, 4H), 1.44 (s, 9H).
^{13}C{^{1}H } NMR (101 MHz, acetone-d_6, 22 °C) δ 169.0 (C), 79.7 (C), 78.8 (C), 73.3 (CH), 59.5 (CH_2), 52.1 (CH_2), 51.3 (CH_2), 46.1 (CH_2), 27.2 (CH_3).

S4：To a solution of **S3** (52.4 mg, 0.220 mmol, 1.0 equiv.) in dried CH_2Cl_2 (1.0 mL) was added trifluoroacetic acid (0.5 mL, d = 1.49 g/mL, 6.53 mmol) at room temperature. The reaction mixture was stirred at room temperature. After 19 h, the resulting solution was concentrated by rotary evaporation. The residue was dissolved in CH_2Cl_2 (5 mL) then concentrated by rotary evaporation (repeated this step three times) to afford a white solid (86.8 mg, 0.230 mmol, quantitative yield). The crude product was directly used to the next step without further purification.

Synthesis of **excB-12*O*-PZyne**

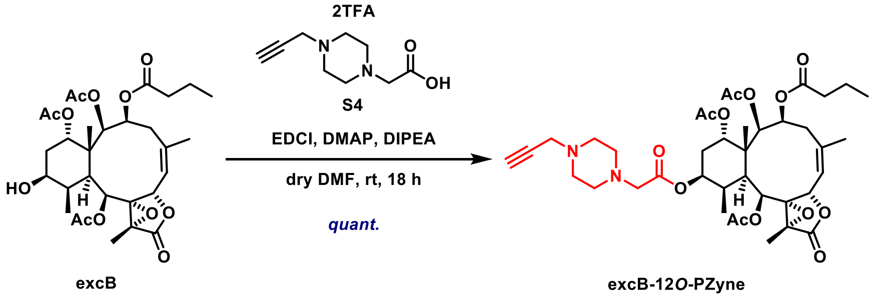

Under an ambient atmosphere, a 4 mL vial equipped with a stir bar was charged with **excB** (33.7 mg, 0.0567 mmol, 1.0 equiv.), **S4** (42.7 mg, 0.113 mmol, 2.0 equiv.), DIPEA (2 drops), DMAP (2.1 mg, 0.017 mmol, 0.3 equiv.), EDCI (32.6 mg, 0.170 mmol, 3.0 equiv.) and dried DMF (0.55 mL). The vial was sealed with a Teflon cap, and the reaction mixture was stirred at room temperature. After 18 h, the reaction mixture was diluted with EtOAc and the organic layer was washed with H_2O. The organic layer was dried over $MgSO_4$, filtered and concentrated by rotary evaporation. The residue was purified by flash column chromatography (EtOAc : *n*-Hexane = 0 : 1→1 : 1→MeOH : CH_2Cl_2 = 1 : 10) to afford **excB-12*O*-PZyne** (47.1 mg, 0.0621 mmol, quantitative yield) as a colorless oil.

TLC (MeOH : CH_2Cl_2 = 1 : 10): R_f = 0.5 ($KMnO_4$).
^{1}H NMR (600 MHz, acetone-d_6, -40 °C) δ 5.87 (dd, *J* = 7.6, 2.6 Hz, 1H), 5.68 (d, *J* = 7.3 Hz, 1H), 5.54 (d, *J* = 10.3 Hz, 1H), 5.42 – 5.35 (m, 1H), 5.18 (d, *J* = 2.4 Hz, 1H), 5.04 (dt, *J* = 12.5, 4.3 Hz, 1H), 4.72 (dd, *J* = 4.0, 2.1 Hz, 1H), 4.03 (dd, *J* = 15.8, 7.3 Hz, 1H), 3.29 (d, *J* = 2.5 Hz, 2H), 3.26, 3.20 (ABq, *J* = 17.0 Hz, 2H), 3.18 (dd, *J* = 17.2, 4.9 Hz, 1H), 2.99 – 2.93 (m, 1H), 2.86 – 2.75 (m, 2H), 2.73 – 2.60 (m, 3H), 2.42 (s, 3H), 2.37 – 2.27 (m, 4H), 2.24 (s, 6H), 2.22 – 2.08 (m, 4H), 1.97 (s, 3H), 1.92 – 1.86 (m, 1H), 1.58 – 1.47 (m, 2H), 1.52 (s, 3H), 1.11 (d, *J* = 7.1 Hz, 3H), 0.87 (s, 3H), 0.87 (t, *J* = 7.4 Hz, 3H).
^{13}C{^{1}H } NMR (151 MHz, acetone-d_6, -40 °C) δ 172.2 (C), 172.1 (C), 171.9 (C), 170.7 (C), 170.1 (C), 169.9 (C), 140.0 (C), 122.6 (CH), 81.4 (2xCH), 79.5 (C), 74.9 (CH), 74.2 (CH), 73.6 (CH), 70.2 (CH), 69.2 (C), 65.3 (CH), 60.6 (C), 59.0 (CH_2), 52.8 (CH_2), 52.7 (CH_2), 51.8 (CH_2), 46.6 (CH_2), 44.2 (C), 40.3 (CH), 35.9 (CH_2), 34.3 (CH_2), 33.0 (CH), 27.2 (CH_2), 22.6 (CH_3), 22.2 (2xCH_3), 21.4 (CH_3), 18.4 (CH_2), 18.0 (CH_3), 13.7 (CH_3), 10.3 (CH_3), 10.0 (CH_3).
HRMS-FAB (m/z) calcd for $C_{39}H_{55}N_2O_{13}$ $[M+H]^+$ 759.3699, found 759.3701.

Synthesis of **excB-16-*CHO***

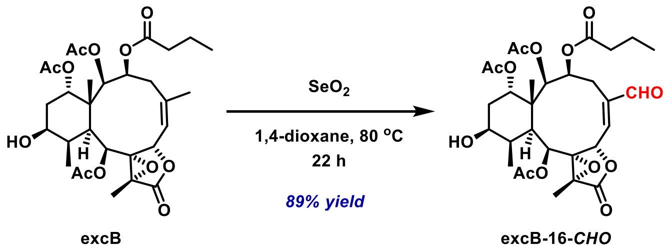

Under an ambient atmosphere, a 4 mL vial equipped with a stir bar was charged with **excB** (54.1 mg, 0.091 mmol, 1.00 equiv.), SeO_2 (100.9 mg, 0.910 mmol, 10.0 equiv.) and 1,4-dioxane (1.0 mL). The vial was sealed with a Teflon cap, and the reaction mixture was stirred at 80 °C. After 22 h, the reaction mixture was cooled to room temperature, filtered through a

short pad of Celite. The filtrate was concentrated under reduced pressure. The residue was purified by flash column chromatography (EtOAc : *n*-Hexane = 0 : 1→2 : 3→1 : 1) to afford **excB-16-*CHO*** (49.4 mg, 0.081 mmol, 89% yield) as a white solid.

TLC (EtOAc : *n*-Hexane = 1 : 1): R_f = 0.30 ($KMnO_4$).
^{1}H NMR (600 MHz, acetone-d_6, -40 °C) δ 9.69 (d, *J* = 1.6 Hz, 1H), 6.88 (dd, *J* = 7.5, 2.1 Hz, 1H), 5.96 (dd, *J* = 7.4, 1.5 Hz, 1H), 5.71 (dd, *J* = 7.6, 2.7 Hz, 1H), 5.64 (d, *J* = 10.4 Hz, 1H), 5.19 (d, *J* = 2.5 Hz, 1H), 4.59 – 4.51 (m, 1H), 4.20 – 4.11 (m, 1H), 3.96 – 3.79 (m, 2H), 2.87 (dd, *J* = 10.4, 5.0 Hz, 1H), 2.57 – 2.50 (m, 1H), 2.46 (d, *J* = 15.1 Hz, 1H), 2.42 (s, 3H), 2.21 (s, 3H), 2.22 – 2.04 (m, 2H), 1.99 (s, 3H), 1.89 – 1.81 (m, 1H), 1.79 – 1.71 (m, 1H), 1.55 (s, 3H), 1.54 – 1.49 (m, 2H), 1.03 (d, *J* = 7.2 Hz, 3H), 0.90 (t, *J* = 7.4 Hz, 3H), 0.79 (s, 3H).
^{13}C{^{1}H } NMR (151 MHz, acetone-d_6, -40 °C) δ 194.9 (CH), 172.2 (C), 171.9 (C), 171.7 (C), 170.8 (C), 170.0 (C), 148.1 (CH), 144.5 (C), 82.0 (CH), 81.3 (CH), 74.6 (CH), 73.9 (CH), 69.9 (C), 65.8 (CH), 65.3 (CH), 60.4 (C), 44.7 (C), 40.8 (CH), 36.0 (CH), 35.9 (CH_2), 29.8 (CH_2), 27.7 (CH_2), 22.7 (CH_3), 22.2 (CH_3), 21.4 (CH_3), 18.5 (CH_2), 18.0 (CH_3), 13.7 (CH_3), 10.0 (CH_3), 9.4 (CH_3).
HRMS-ESI (m/z) calcd for $C_{30}H_{40}O_{14}Na$ $[M+Na]^+$ 647.2370, found 647.2361.

Synthesis of **excB-16*C*-AO**[4]

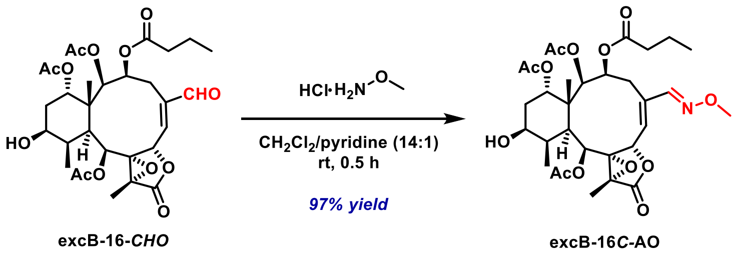

Under an ambient atmosphere, a 4 mL vial equipped with a stir bar was charged with **excB-16-*CHO*** (18.2 mg, 0.0299 mmol, 1.0 equiv.), *O*-methylhydroxylamine hydrochloride (3.8 mg, 0.0449 mmol, 1.5 equiv.), CH_2Cl_2/pyridine (0.3 mL, ratio 14 : 1). The vial was sealed with a Teflon cap, and the reaction mixture was stirred at room temperature. After 0.5 h, the reaction mixture was directly purified by flash column chromatography (EtOAc : *n*-Hexane = 0 : 1→1 : 1) to afford **excB-16*C*-AO** (18.4 mg, 0.0289 mmol, 97% yield) as a colorless oil.

TLC (EtOAc : *n*-Hexane = 1 : 1): R_f = 0.23 (UV_{254}, $KMnO_4$).
^{1}H NMR (600 MHz, acetone-d_6, -40 °C) δ 7.97 (s, 1H), 6.06 – 5.99 (m, 1H), 5.92 (d, *J* = 6.0 Hz, 1H), 5.83 (d, *J* = 7.4 Hz, 1H), 5.61 (d, *J* = 10.3 Hz, 1H), 5.17 (s, 1H), 4.58 (s, 1H), 4.22 – 4.15 (m, 1H), 3.97 (dd, *J* = 15.7, 7.3 Hz, 1H), 3.91 – 3.83 (m, 1H), 3.75 (s, 3H), 2.98 (dd, *J* = 10.4, 5.0 Hz, 1H), 2.57 – 2.48 (m, 2H), 2.41 (s, 3H), 2.21 (s, 3H), 2.19 – 2.08 (m, 2H), 2.05 (s, 3H), 1.91 – 1.80 (m, 1H), 1.80 – 1.73 (m, 1H), 1.58 – 1.46 (m, 2H), 1.53 (s, 3H), 1.04 (d, *J* = 7.2 Hz, 3H), 0.89 (t, *J* = 7.4 Hz, 3H), 0.81 (s, 3H).
^{13}C{^{1}H } NMR (151 MHz, acetone-d_6, -40 °C) δ 172.2 (C), 172.1 (C), 171.7 (C), 170.7 (C), 169.9 (C), 150.1 (CH), 138.1 (C), 133.2 (CH), 82.0 (CH), 81.5 (CH), 75.4 (CH), 74.0 (CH), 69.7 (C), 65.8 (CH), 65.4 (CH), 61.9 (CH_3), 60.5 (C), 44.6 (C), 40.6 (CH), 36.1 (CH), 36.0 (CH_2), 29.8 (CH_2), 29.2 (CH_2), 22.7 (CH_3), 22.2 (CH_3), 21.4 (CH_3), 18.6 (CH_2), 18.1 (CH_3), 13.9 (CH_3), 10.0 (CH_3), 9.5 (CH_3).
HRMS-ESI (m/z) calcd for $C_{31}H_{43}NO_{13}Na$ $[M+Na]^+$ 660.2627, found 660.2634.

Synthesis of **excB-16*C*-AOyne**[4]

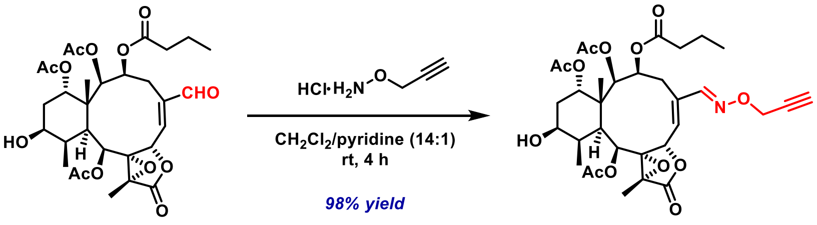

excB-16-*CHO* excB-16*C*-AOyne

Under an ambient atmosphere, a 4 mL vial equipped with a stir bar was charged with **excB-16-*CHO*** (50.5 mg, 0.0830 mmol, 1.0 equiv.), *O*-(prop-2-yn-1-yl)hydroxylamine hydrochloride (13.4 mg, 0.1245 mmol, 1.5 equiv.), CH_2Cl_2/Pyridine (1.0 mL, ratio 14 : 1). The vial was sealed with a Teflon cap, and the reaction mixture was stirred at room temperature. After 4 h, the reaction mixture was directly purified by flash column chromatography (EtOAc : *n*-Hexane = 0 : 1→2 : 3→1 : 1) to afford **excB-16*C*-AOyne** (54.0 mg, 0.0816 mmol, 98% yield) as a colorless oil.

TLC (EtOAc : *n*-Hexane = 1 : 1): R_f = 0.25 (UV_{254}, $KMnO_4$).
^{1}H NMR (600 MHz, acetone-d_6, -40 °C) δ 8.04 (s, 1H), 6.09 (dd, *J* = 7.5, 2.0 Hz, 1H), 5.92 (dd, *J* = 6.6, 2.0 Hz, 1H), 5.84 (d, *J* = 7.4 Hz, 1H), 5.62 (d, *J* = 10.4 Hz, 1H), 5.16 (d, *J* = 2.5 Hz, 1H), 4.65 (dd, *J* = 15.8, 2.5 Hz, 1H), 4.61 – 4.55 (m, 2H), 4.17 (d, *J* = 4.0 Hz, 1H), 4.01 (dd, *J* = 15.6, 6.7 Hz, 1H), 3.91 – 3.84 (m, 1H), 3.26 (t, *J* = 2.4 Hz, 1H), 2.96 (dd, *J* = 10.4, 5.1 Hz, 1H), 2.58 – 2.49 (m, 2H), 2.41 (s, 3H), 2.21 (s, 3H), 2.17 – 2.05 (m, 2H), 2.05 (s, 3H), 1.90 – 1.80 (m, 1H), 1.79 – 1.72 (m, 1H), 1.58 – 1.48 (m, 2H), 1.53 (s, 3H), 1.04 (d, *J* = 7.3 Hz, 3H), 0.89 (t, *J* = 6.0 Hz, 3H), 0.81 (s, 3H).
^{13}C{^{1}H } NMR (151 MHz, acetone-d_6, -40 °C) δ 172.2 (C), 172.0 (C), 171.9 (C), 170.7 (C), 169.9 (C), 151.2 (CH), 137.8 (C), 134.2 (CH), 82.0 (CH), 81.6 (CH), 79.9 (C), 76.7 (CH), 75.2 (CH), 74.0 (CH), 69.7 (C), 65.8 (CH), 65.4 (CH), 61.9 (CH_2), 60.5 (C), 44.6 (C), 40.6 (CH), 36.1 (CH), 36.1 (CH_2), 30.1 (CH_2), 29.2 (CH_2), 22.7 (CH_3), 22.3 (CH_3), 21.4 (CH_3), 18.5 (CH_2), 18.0 (CH_3), 13.9 (CH_3), 10.0 (CH_3), 9.5 (CH_3).
HRMS-FAB (m/z) calcd for $C_{33}H_{43}NO_{13}Na$ $[M+Na]^+$ 684.2627, found 684.2620.

Synthesis of (*E*)-2-((2*E*,4*E*)-5-(1-(1-Azido-13-oxo-3,6,9-trioxa-12-azaoctadecan-18-yl)-3,3-dimethyl-5-sulfo-3H-indol-1-ium-2-yl)penta-2,4-dien-1-ylidene)-1,3,3-trimethylindoline-5-sulfonate (azide-Cy5)**

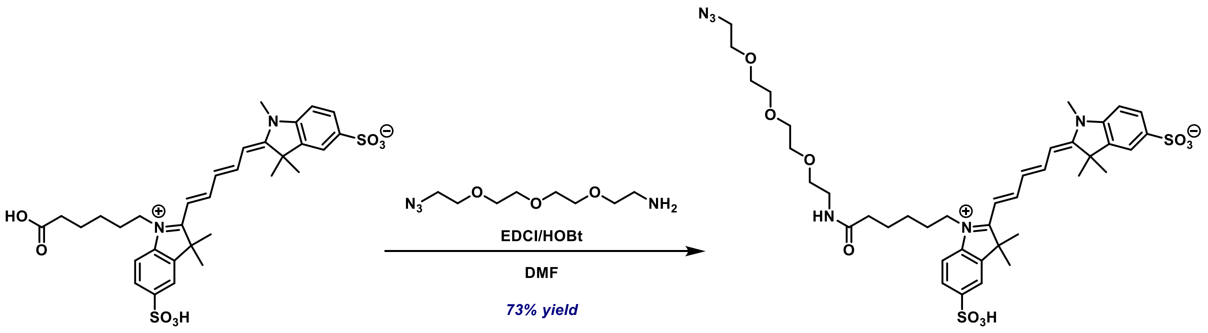

Under an ambient atmosphere, a 4 mL vial equipped with a stir bar was charged with sulfo-cyanine5 carboxylic acid (10.0 mg, 0.015 mmol, 1.1 equiv.) in DMF (1 mL) was added EDCI (5.0 mg, 0.032 mmol, 2.1 equiv.) and HOBt (5.0 mg, 0.037 mmol, 2.4 equiv.) were kept in dark and stirred for 1 h at room temperature. After 1 h, a solution of 11-azido-3,6,9-trioxaundecan-1-amine (10 mg, 0.045 mmol, 3.0 equiv.) in DMF (1.0 mL) was then added to the above solution at the same temperature. After stirring for additional 15 h, ether was added to the reaction mixture and filtered to collect the precipitate. The residue was purified by flash chromatography column (MeOH/H_2O = 0% to 35%, RP-18 silica gel) to afford **azide-Cy5** (10 mg, 0.011 mmol, 73% yield) as yellowish solid.

^{1}HNMR (600 MHz, MeOD) δ 8.40 – 8.22 (m, 1H), 8.02 – 7.73 (m, 2H), 7.34 (dd, *J* = 8.1, 1.0 Hz, 1H), 6.68 (t, *J* = 12.4 Hz, 1H), 6.34 (dd, *J* = 13.7, 6.5 Hz, 1H), 4.13 (t, *J* = 7.4 Hz, 1H), 3.79 – 3.55 (m, 6H), 3.52 (t, *J* = 5.5 Hz, 1H), 3.45 – 3.38 (m, 1H), 3.35 (ddd, *J* = 4.8, 3.9, 2.1 Hz, 2H), 3.00 – 2.90 (m, 1H), 2.40 (t, *J* = 6.6 Hz, 1H), 2.28 (s, 1H), 2.22 (t, *J* = 7.3 Hz, 1H), 1.84 (p, *J* = 8.0 Hz, 1H), 1.79 – 1.74 (m, 3H), 1.74 – 1.65 (m, 1H), 1.47 (p, *J* = 7.8 Hz, 1H), 1.31 (d, *J* = 13.3 Hz, 1H), 1.25 (t, *J* = 7.2 Hz, 1H).
HRMS (ESI): calc. for $C_{40}H_{55}N_6O_{10}S_2^+$: 843.3416, found: 843.3404.

Supplementary References

1. Livak, K. J. & Schmittgen, T. D. Analysis of relative gene expression data using real-time quantitative PCR and the 2(-Delta Delta C(T)) Method. *Methods* **25**, 402–408 (2001).
2. Bryan, N. S. & Grisham, M. B. Methods to detect nitric oxide and its metabolites in biological samples. *Free Radic Biol Med* **43**, 645–657 (2007).
3. Robbins, D. W., Peng, G., Mihalic, J. & Sands, A. T. Bifunctional compounds for degrading btk via ubiquitin proteosome pathway. (2021).
4. Nakayama, Y. *et al.* Total synthesis of ritterazine B. *J. Am. Chem. Soc.* **143**, 4187–4192 (2021).
