# Supplementary material for: Marine diterpenoid targets STING palmitoylation in mammalian cells
Source: Commun Chem. 2023 Jul 18;6:153. doi: 10.1038/s42004-023-00956-9 (PMC10354091; doi:10.1038/s42004-023-00956-9)
Supplement: Supplementary file 2 — Description of Additional Supplementary Files [file 42004_2023_956_MOESM2_ESM.pdf]

Description of Additional Supplementary Files

File name: Supplementary Data 1

Description: NMR spectra

File name: Supplementary Data 2

Description: List of candidate cellular targets of excB identified from RAW 264.7 macrophages

File name: Supplementary Data 3

Description: Primers used in this study

File name: Supplementary Data 4

Description: Source data and specific data P-values
