# Supplementary figures and images for: Marine diterpenoid targets STING palmitoylation in mammalian cells
Source: Commun Chem. 2023 Jul 18;6:153. doi: 10.1038/s42004-023-00956-9 (PMC10354091; doi:10.1038/s42004-023-00956-9)

**Supplementary Data 1. NMR spectra.**


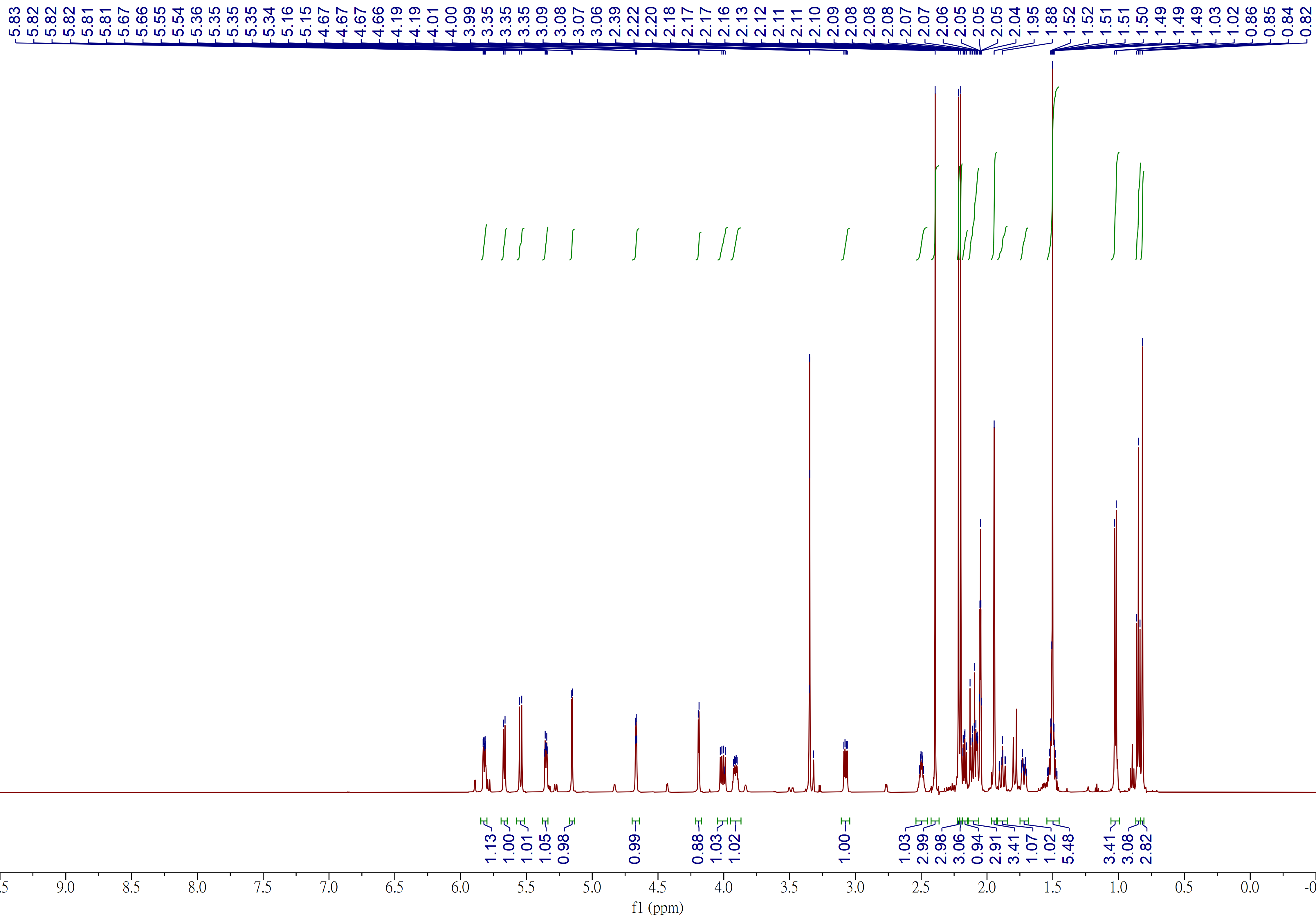


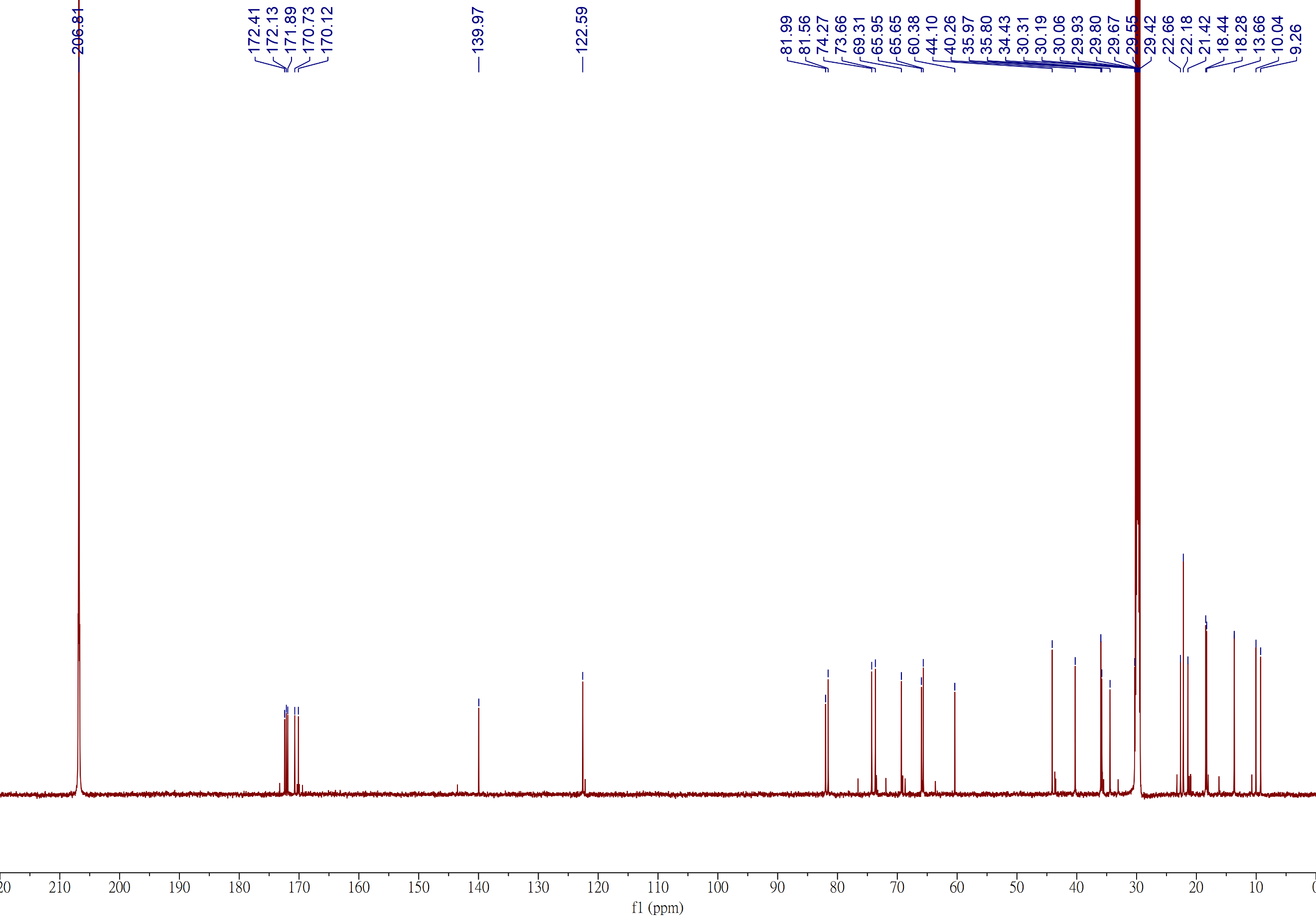


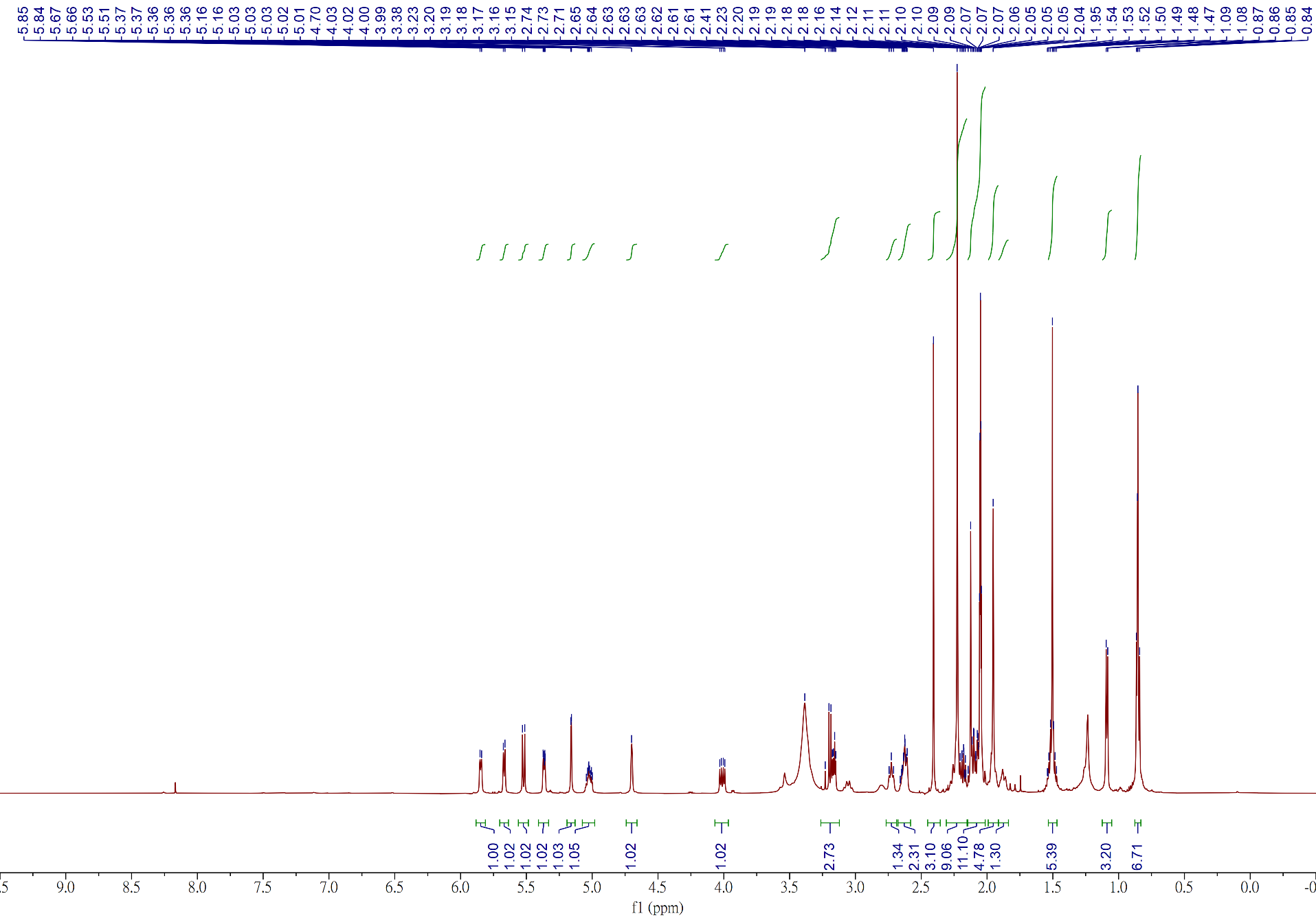


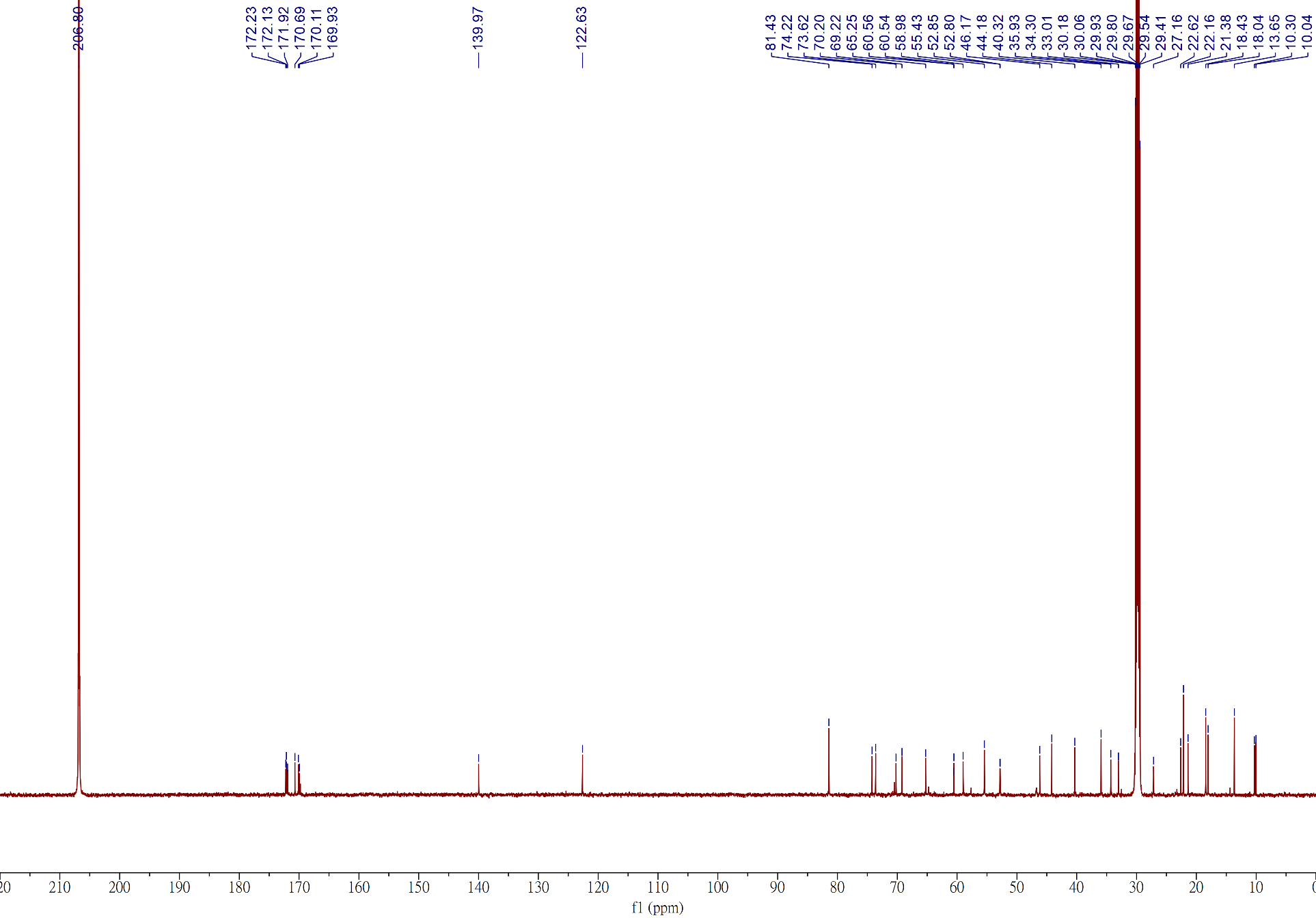


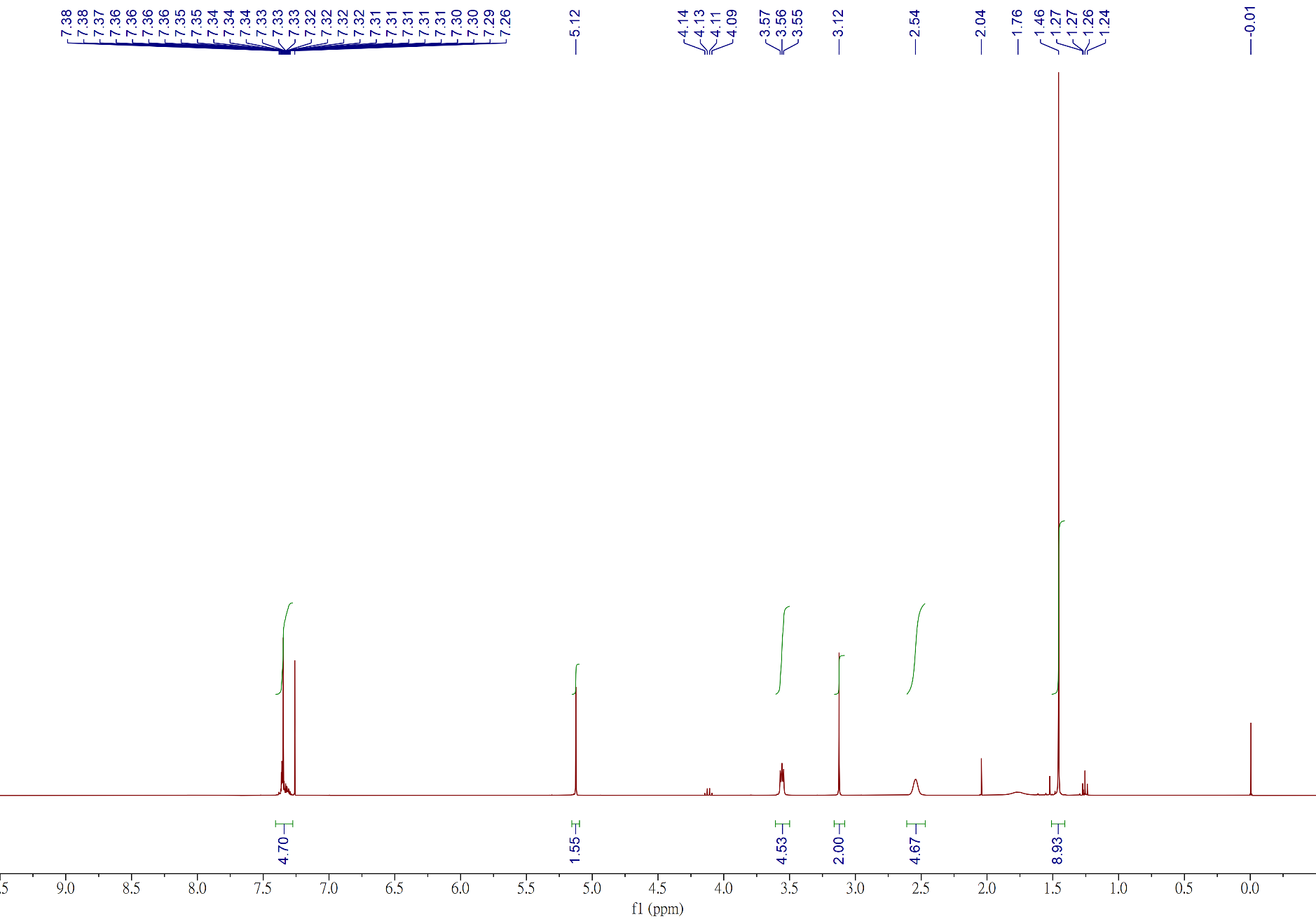


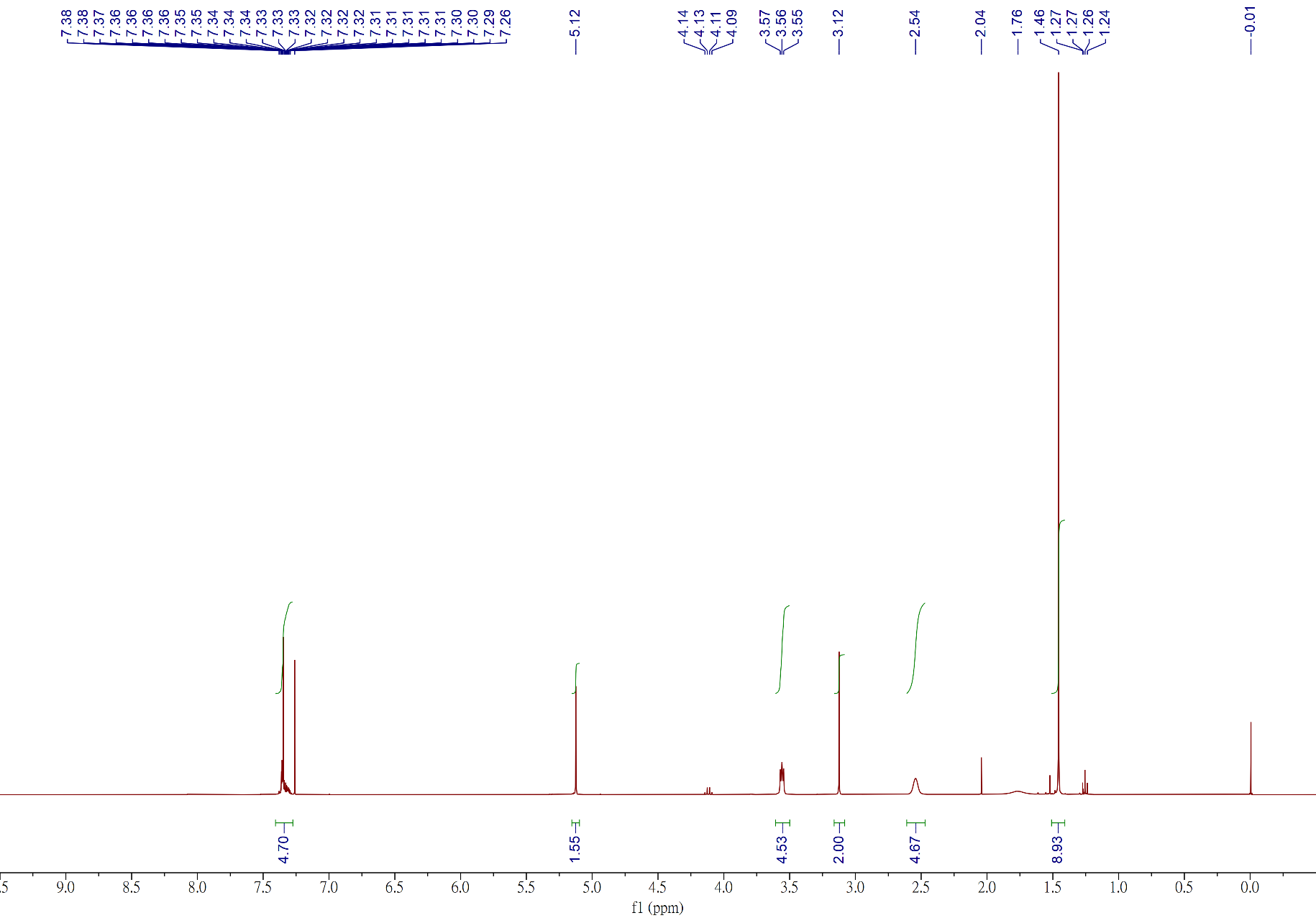


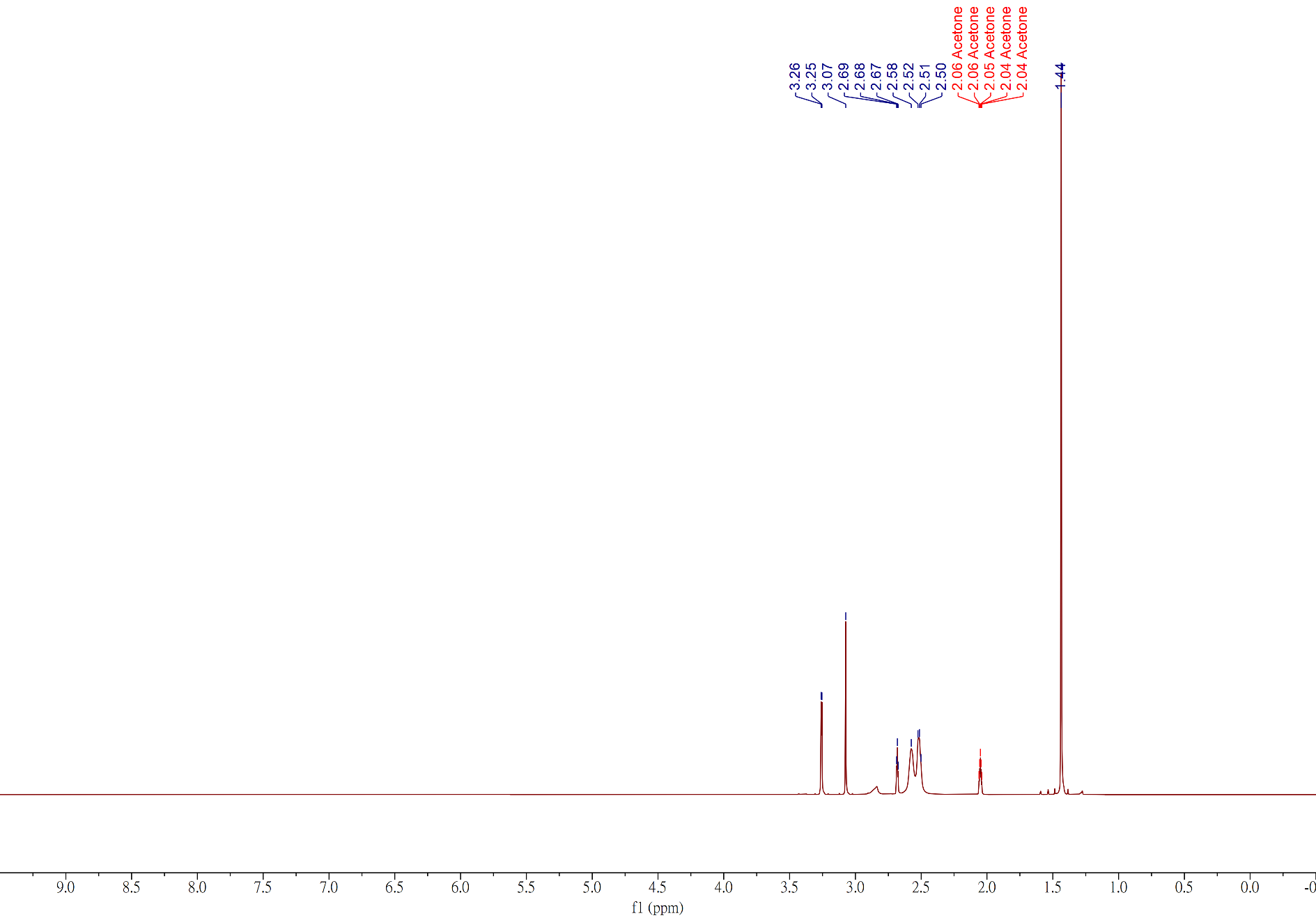


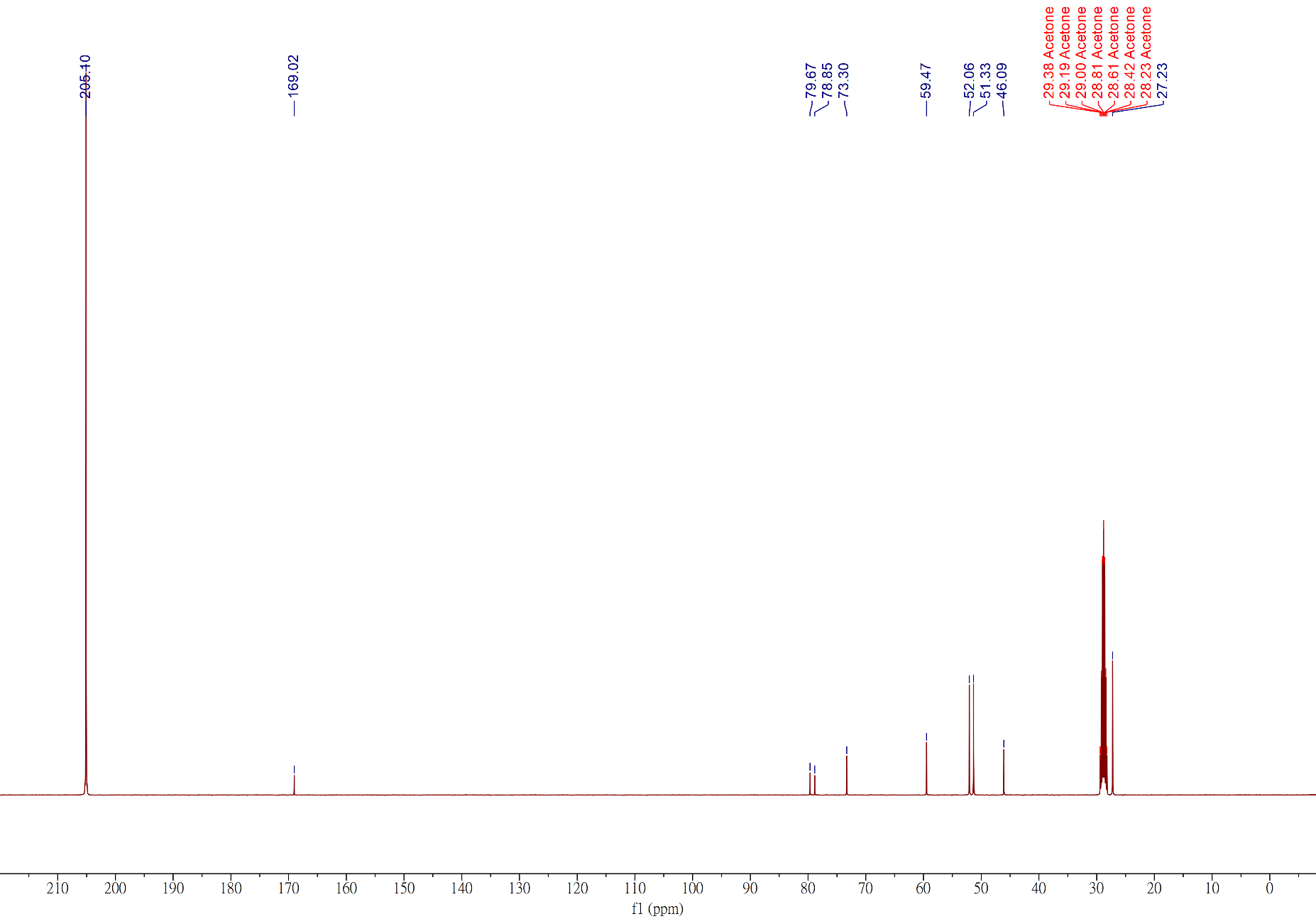


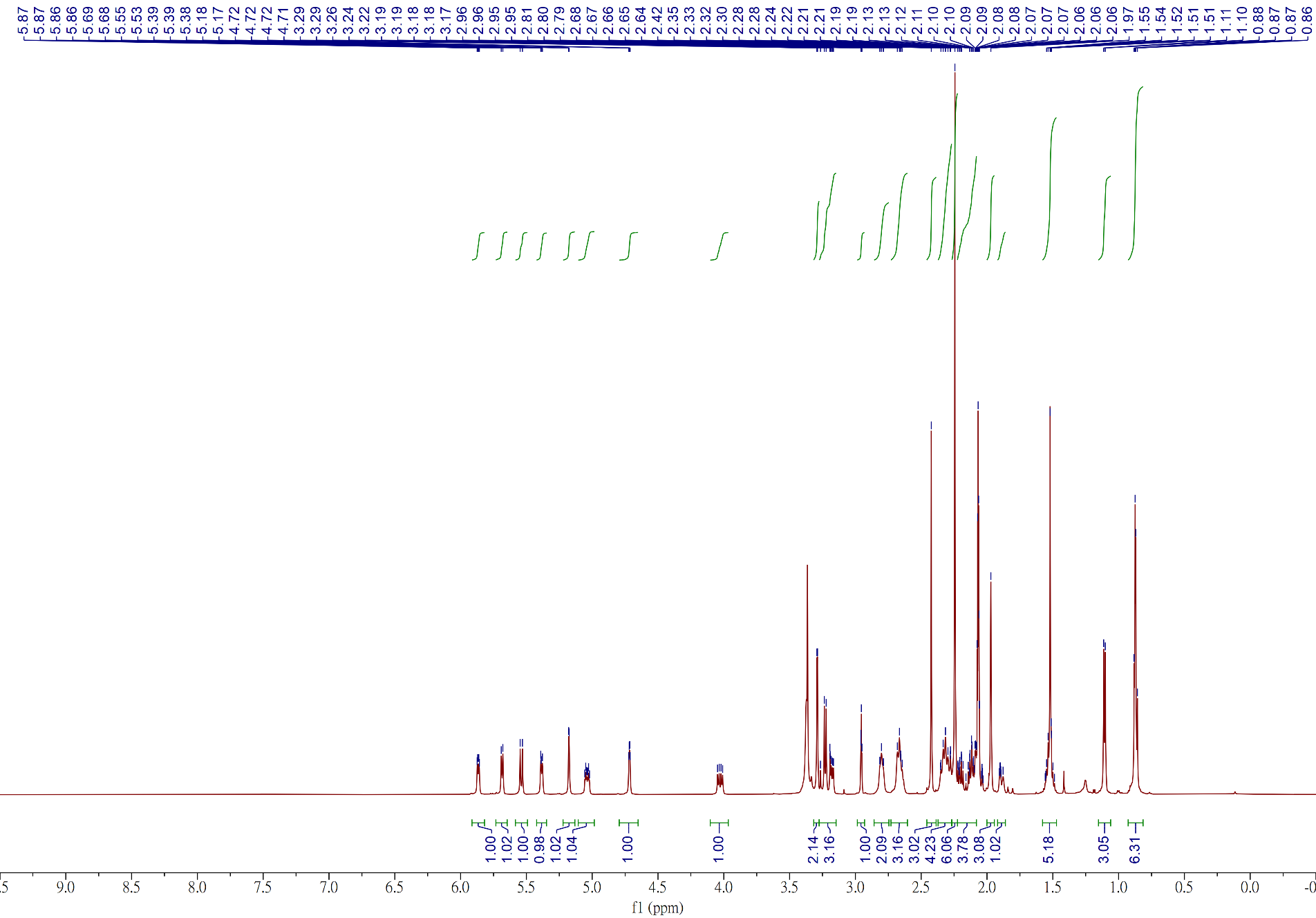


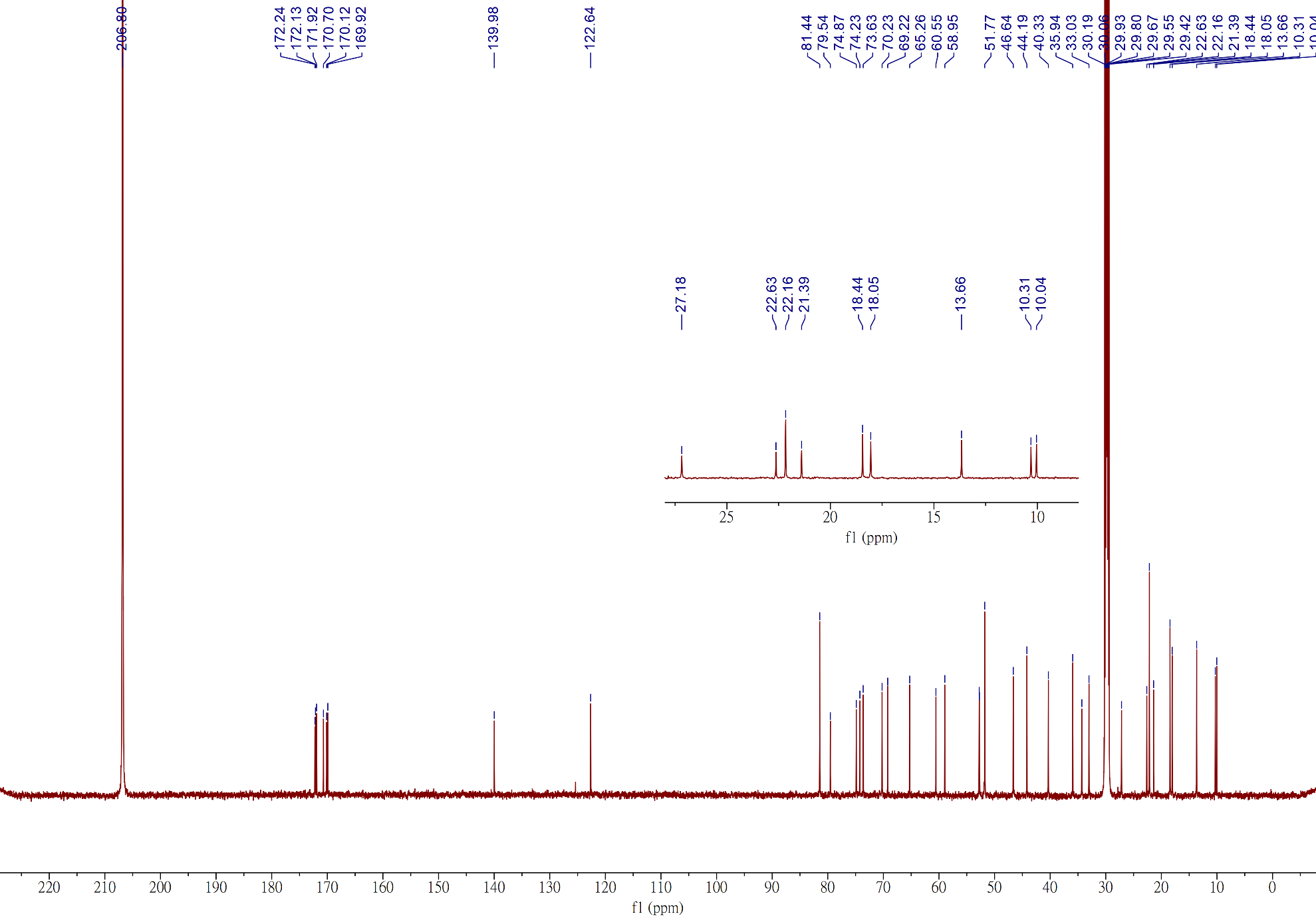


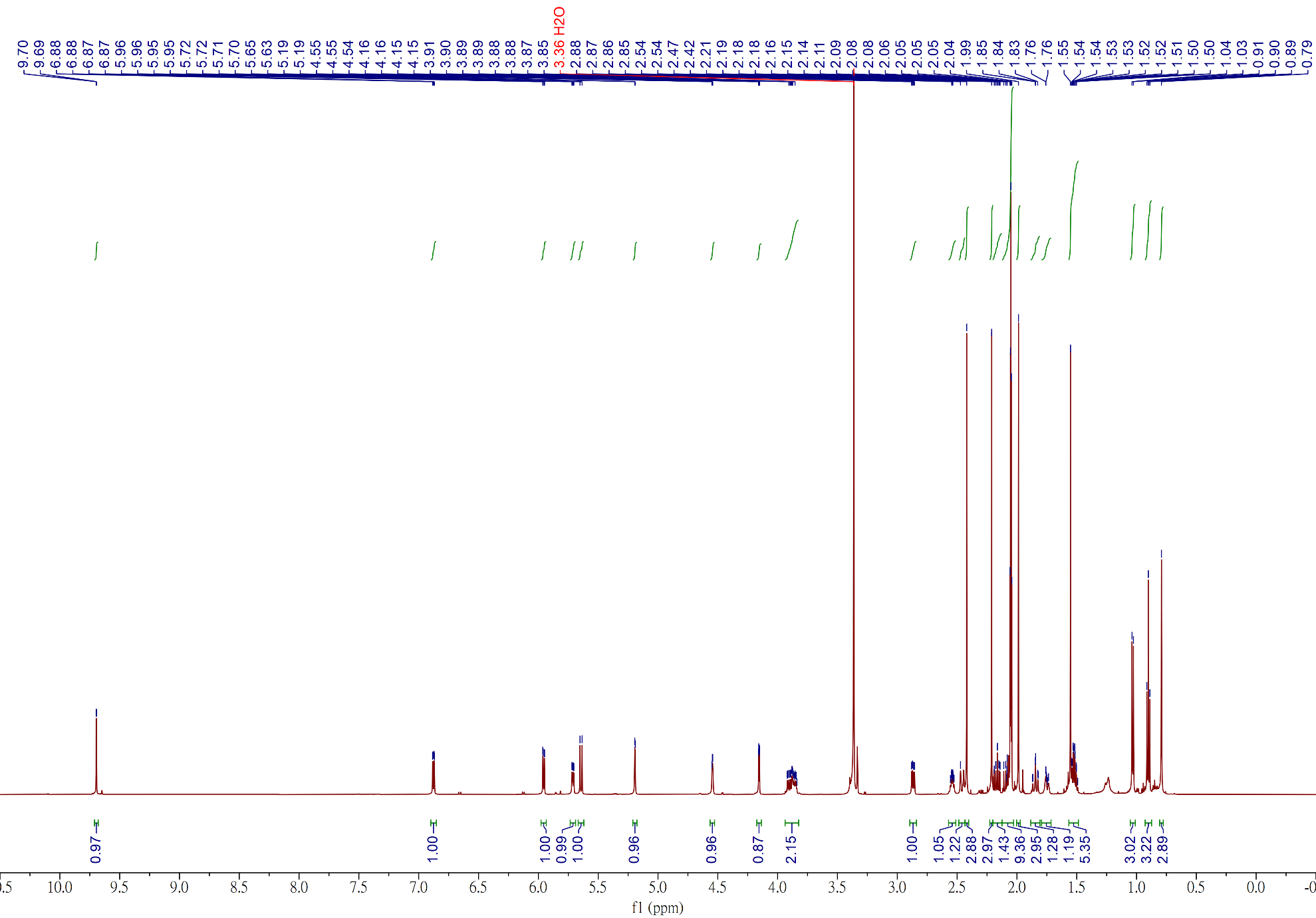


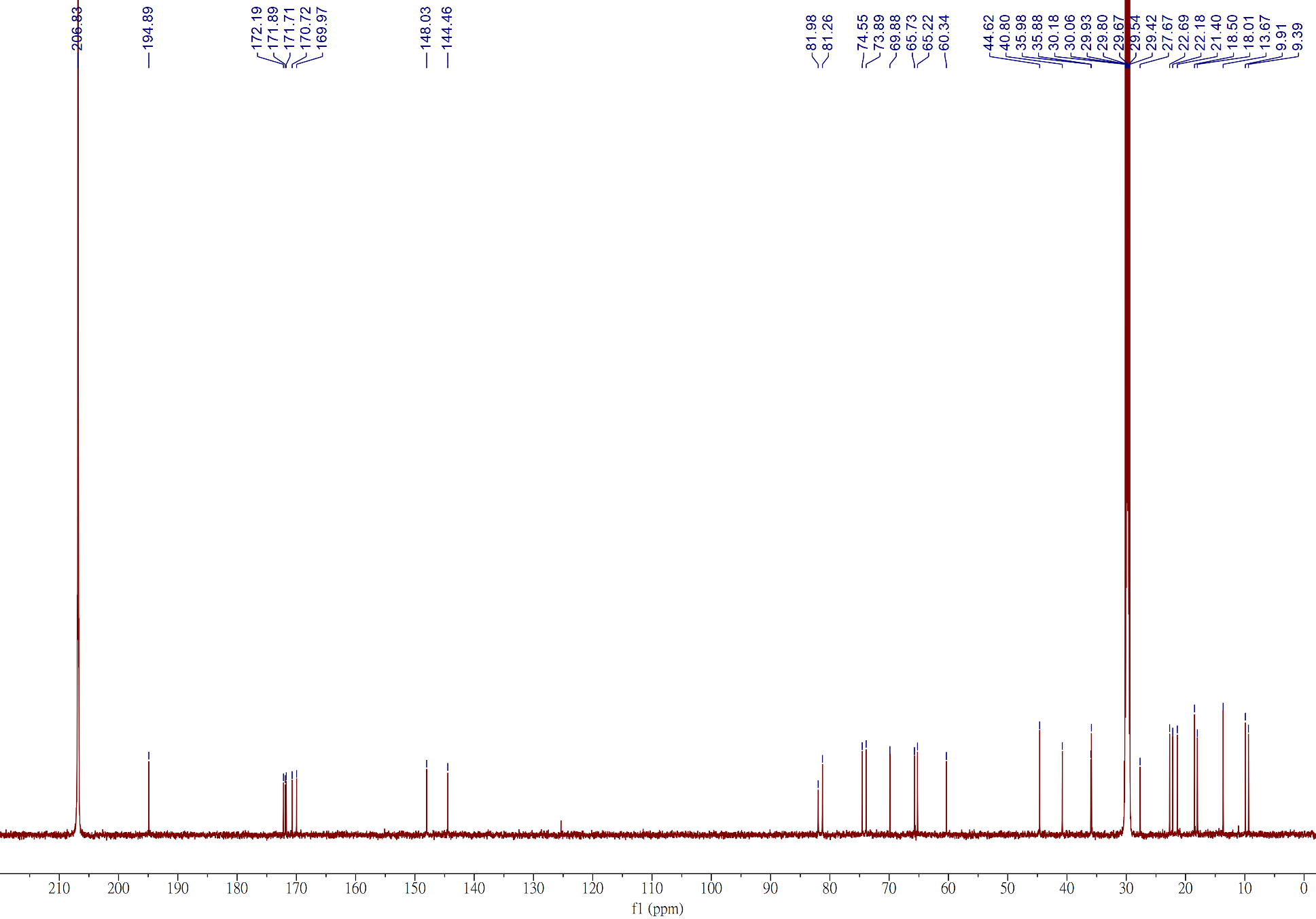


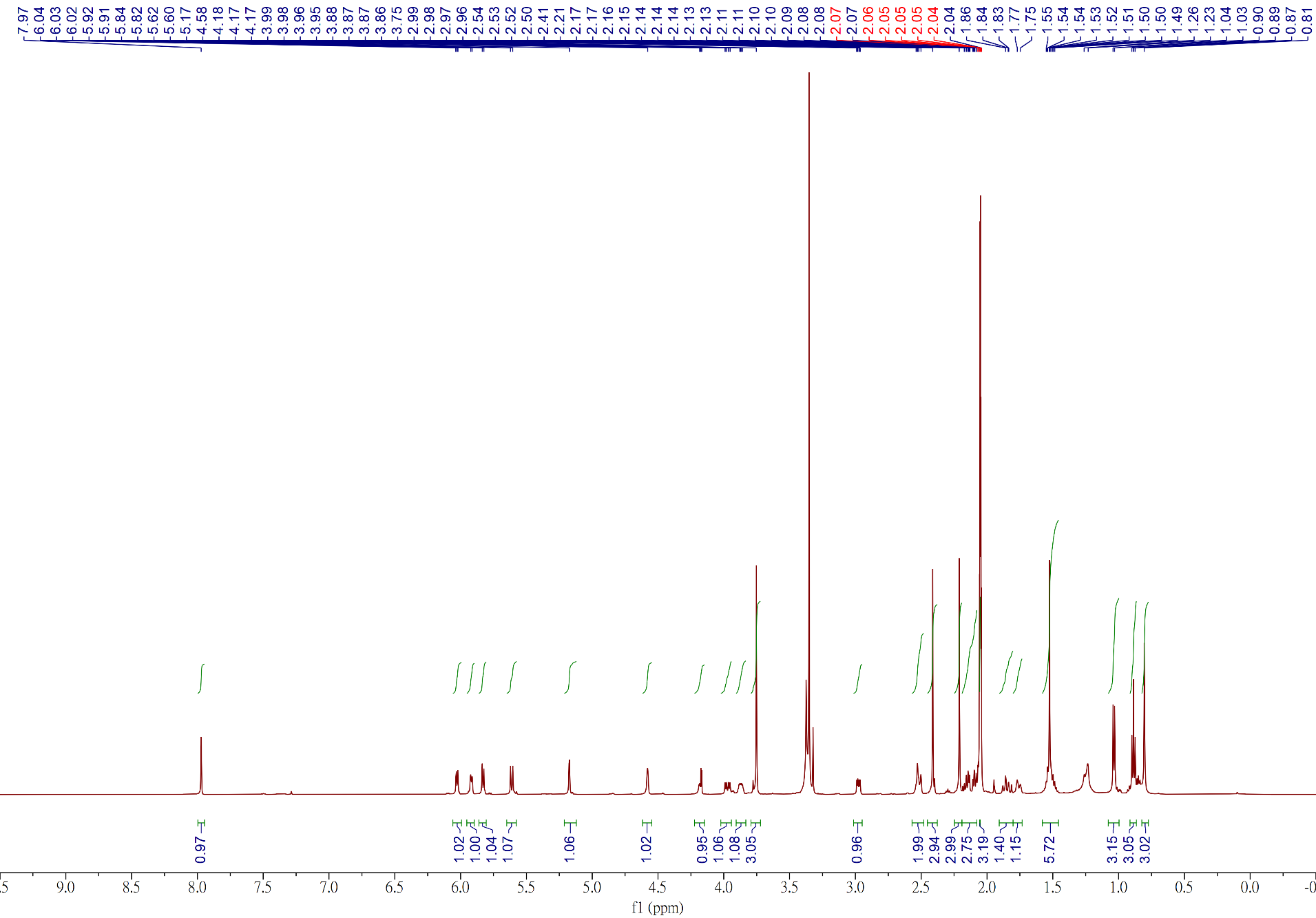


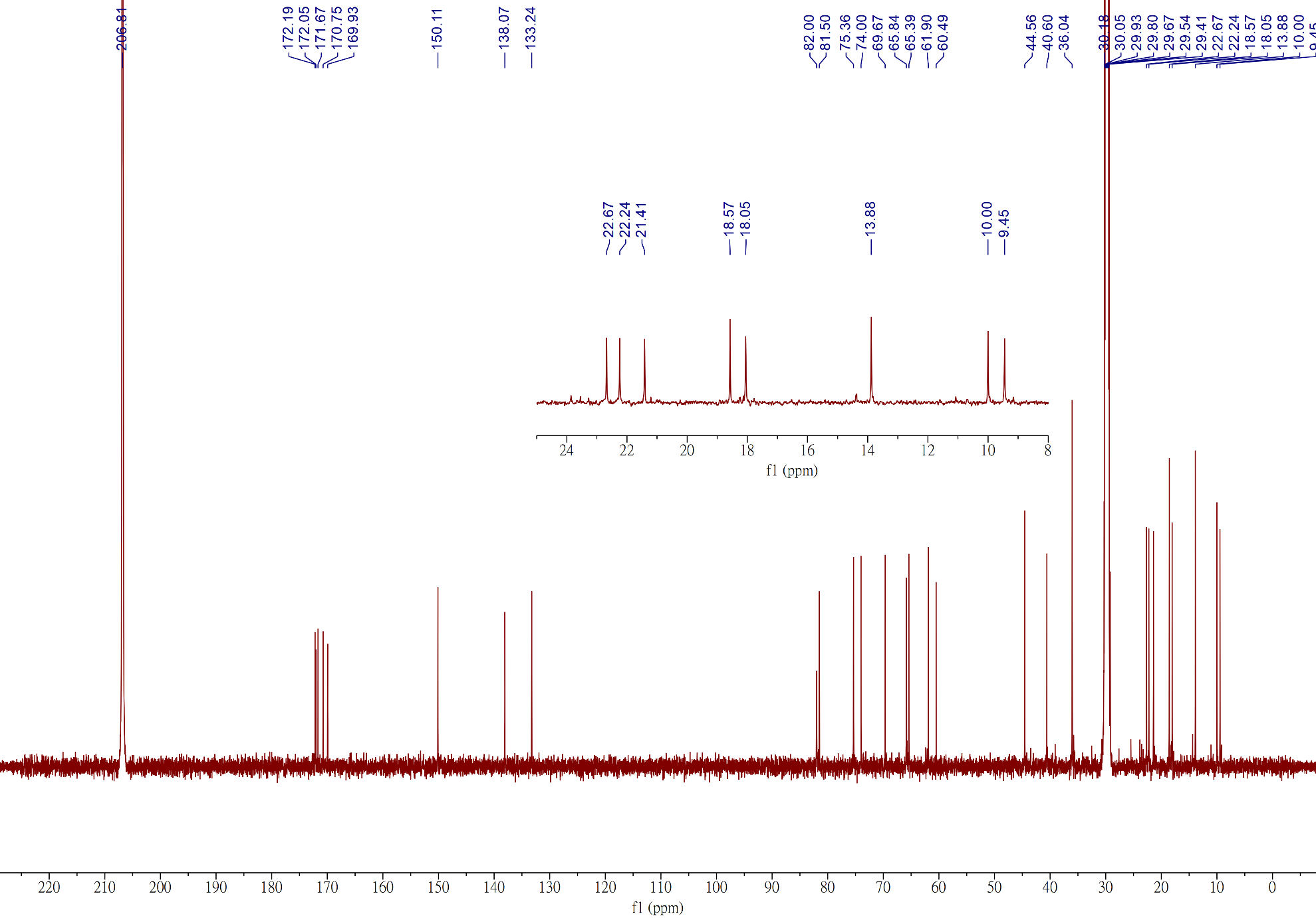


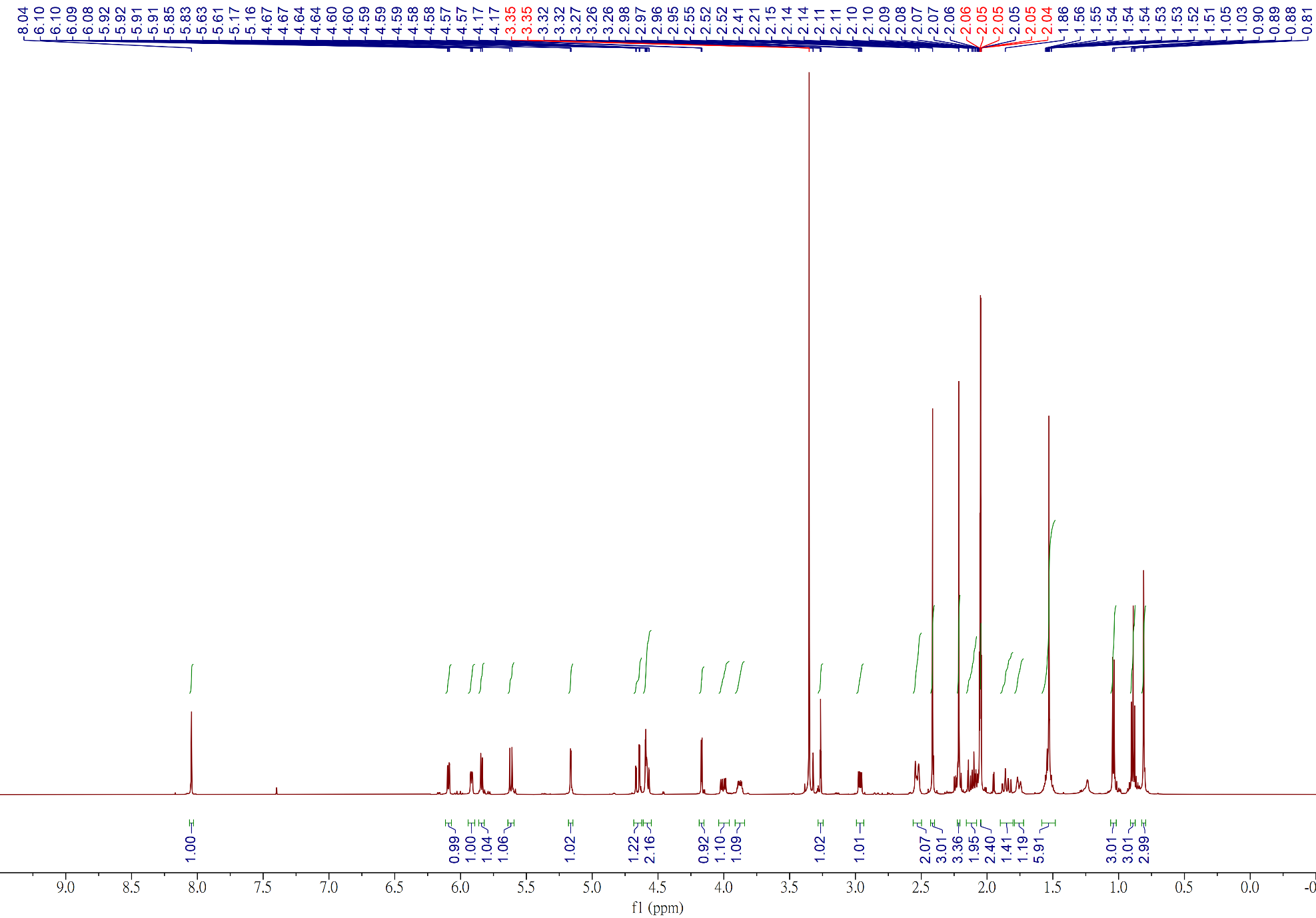


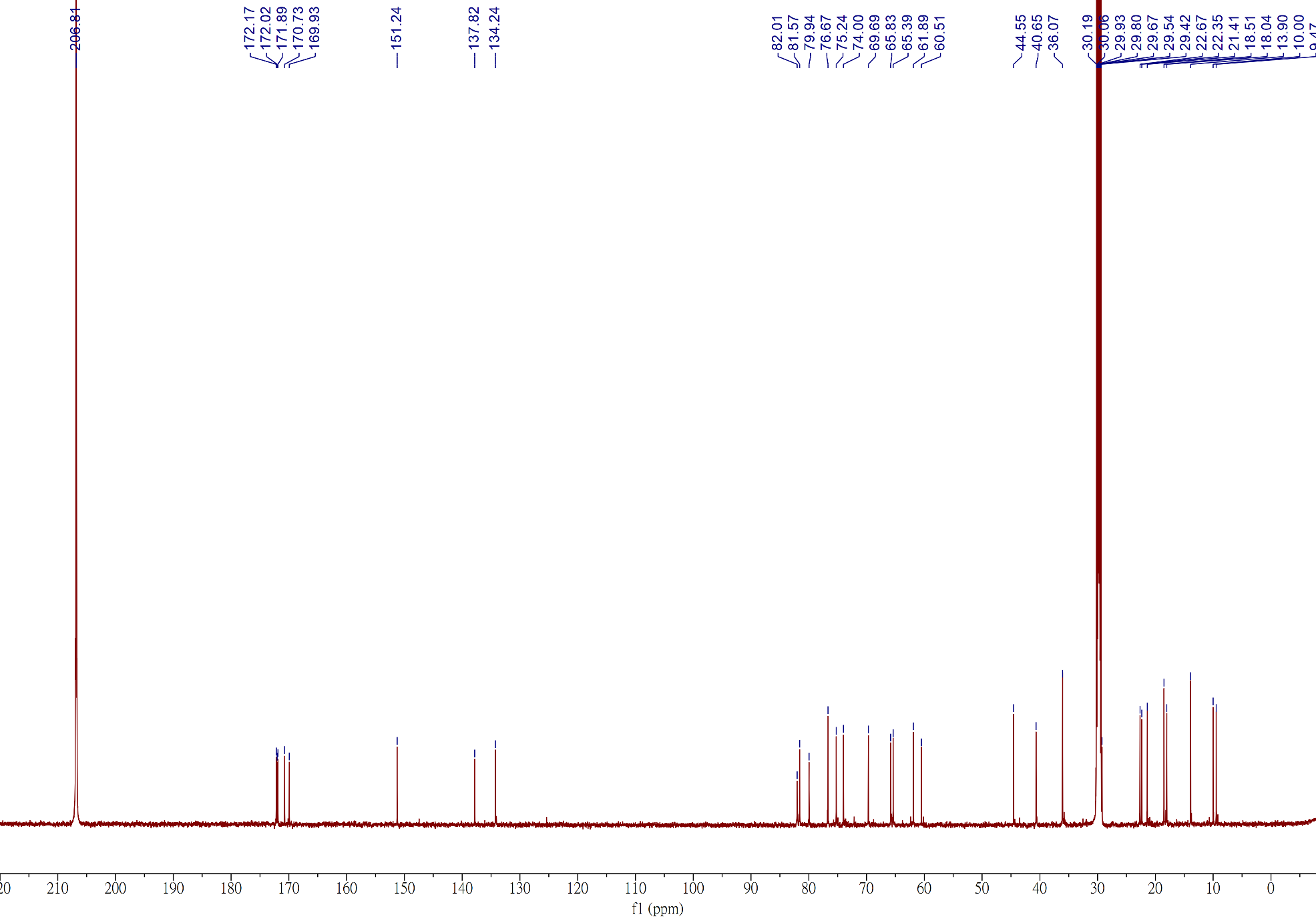

Supplement: Supplementary file 3 — Supplementary Data 1 [file 42004_2023_956_MOESM3_ESM.docx]
