# Supplementary material for: Marine diterpenoid targets STING palmitoylation in mammalian cells
Source: Commun Chem. 2023 Jul 18;6:153. doi: 10.1038/s42004-023-00956-9 (PMC10354091; doi:10.1038/s42004-023-00956-9)
Supplement: Supplementary file 7 — Reporting Summary [file 42004_2023_956_MOESM7_ESM.pdf]

nature portfolio

Corresponding author(s): Ping-Jyun Sung
Lun Kelvin Tsou
Mingzi M. Zhang

Last updated by author(s): Jun 28, 2023

Reporting Summary

Nature Portfolio wishes to improve the reproducibility of the work that we publish. This form provides structure for consistency and transparency in reporting. For further information on Nature Portfolio policies, see our Editorial Policies and the Editorial Policy Checklist.

Statistics

For all statistical analyses, confirm that the following items are present in the figure legend, table legend, main text, or Methods section.

n/a	Confirmed	
☐	☒	The exact sample size (*n*) for each experimental group/condition, given as a discrete number and unit of measurement
☐	☒	A statement on whether measurements were taken from distinct samples or whether the same sample was measured repeatedly
☐	☒	The statistical test(s) used AND whether they are one- or two-sided<br>*Only common tests should be described solely by name; describe more complex techniques in the Methods section.*
☒	☐	A description of all covariates tested
☐	☒	A description of any assumptions or corrections, such as tests of normality and adjustment for multiple comparisons
☐	☒	A full description of the statistical parameters including central tendency (e.g. means) or other basic estimates (e.g. regression coefficient) AND variation (e.g. standard deviation) or associated estimates of uncertainty (e.g. confidence intervals)
☐	☒	For null hypothesis testing, the test statistic (e.g. F, t, r) with confidence intervals, effect sizes, degrees of freedom and P value noted<br>*Give P values as exact values whenever suitable.*
☒	☐	For Bayesian analysis, information on the choice of priors and Markov chain Monte Carlo settings
☒	☐	For hierarchical and complex designs, identification of the appropriate level for tests and full reporting of outcomes
☒	☐	Estimates of effect sizes (e.g. Cohen's *d*, Pearson's *r*), indicating how they were calculated

Our web collection on statistics for biologists contains articles on many of the points above.

Software and code

Policy information about availability of computer code

Data collection	NMR data of synthesized compounds were collected on a Bruker Ascend 400 spectrometer operating at 400 MHz for 1H and 100 MHz for 13C, Bruker Ascend 600 operating at 600 MHz for 1H and 151 MHz for 13C acquisitions, respectively. Electrospray mass spectra (ESMS) were recorded as m/z values using an Agilent 1290 infinity II mass spectrometer. High-resolution mass spectra (HRMS) were obtained on JEOL JMS-700 (FAB) or Waters LCT (ESI). SDS-PAGE for in-gel fluorescence and coomassie blue stain and Western blot were imaged on the Bio-Rad ChemiDoc MP imaging system. Proteomics data were collected on an Orbitrap Elite hybrid mass spectrometer (Thermo Electron). The protein-ligand docking data in this study were collected using reported and open source codes for AutoDock Vina 1.2.3, AutoDock4, AutoDockFR 1.0, CB-Dock 1.0, and open source for Python 3.7 and RDKit 2022.9.4 library. The codes and data were deposited on GitHub with the Zenodo DOI: 10.5281/zenodo.7993805. Intact mass spectrometry data was collected on a Thermo Q-Exactive Plus mass spectrometer in high mass range mode. Luciferase reporter assay was performed on Perkin Elmer Wallac Victor2 1420 Multilabel Counter. RT-qPCR data was collected on the ViiA7 Real-Time PCR Systems (Applied Biosystems). Cell-based MTS, nitric oxide and IL-6 production assays were performed using the TECAN Infinite 200 pro spectrophotometer. Experimental details of data collection were described in Methods section.
Data analysis	MestReNova (v14.2.2) was used for NMR spectrum analyses. Image Lab v5.2 (BioRad) was used for gel analysis. Proteomics data were processed by Proteome Discoverer (v2.5.0; Thermo Scientific, Waltham, MA, USA) and searched against Swiss-Prot protein sequence database and cRAP contaminant database with the Mascot server (v2.8.0; Matrix Science, Boston, MA, USA). Intact mass data were deconvoluted using the Intact Protein Analysis in BioPharma Finder v3.2 software. The protein-ligand binding pose analysis and figure generation were done using commercial PyMOL v2.5.4 software. RT-qPCR results were analyzed with the ViiA7 software (Applied Biosystems). GraphPad Prism v8.4.2 was used for analyses of cell-based assays and statistical tests.

For manuscripts utilizing custom algorithms or software that are central to the research but not yet described in published literature, software must be made available to editors and reviewers. We strongly encourage code deposition in a community repository (e.g. GitHub). See the Nature Portfolio guidelines for submitting code & software for further information.

Data

Policy information about availability of data

All manuscripts must include a data availability statement. This statement should provide the following information, where applicable:

- Accession codes, unique identifiers, or web links for publicly available datasets
- A description of any restrictions on data availability
- For clinical datasets or third party data, please ensure that the statement adheres to our policy

The mass spectrometry proteomics data have been deposited to the ProteomeXchange Consortium via the PRIDE partner repository with the dataset identifier PXD042748. The molecular docking codes and data have been deposited on GitHub with Zenodo DOI: 10.5281/zenodo.7993805. NMR data and primers are included in the Supplementary Data. Source data are provided with this paper as Supplementary Data 4. Specific data P-values are also included within Supplementary Data 4.

Human research participants

Policy information about studies involving human research participants and Sex and Gender in Research.

Reporting on sex and gender	This study did not involve any human research participants.
Population characteristics	This study did not involve any human research participants.
Recruitment	This study did not involve any human research participants.
Ethics oversight	This study did not involve any human research participants.

Note that full information on the approval of the study protocol must also be provided in the manuscript.

Field-specific reporting

Please select the one below that is the best fit for your research. If you are not sure, read the appropriate sections before making your selection.

☒ Life sciences ☐ Behavioural & social sciences ☐ Ecological, evolutionary & environmental sciences

For a reference copy of the document with all sections, see nature.com/documents/nr-reporting-summary-flat.pdf

Life sciences study design

All studies must disclose on these points even when the disclosure is negative.

Sample size	Chemical proteomics samples have three independent biological repeats for each condition/treatment. For iNOS gene expression assay, samples for each condition have 3 technical replicates and three independent biological repeats. For nitric oxide production assay, samples for each condition have 1-2 technical assays over three independent biological repeats. For IL-6 production assay, samples for each condition were single-well assays over two independent biological repeats. For cell viability assay, samples for each condition have 3 technical replicates and three independent biological repeats. For IFN assay, samples for each condition were single-well assays over two independent biological repeats. At least independent two biological repeats were performed for SDS-PAGE, in-gel fluorescence and Western Blot experiments unless indicated otherwise. For Figures 3d and 5a, detection by Western blot was performed only once given that there are at least two independent biological repeats of the same conditions with in-gel fluorescence detection (overall at least three independent biological repeats with two detection methods). Chemical syntheses were carried out as single experiments.
Data exclusions	No data was excluded from the analysis.
Replication	The number of biological repeats are noted in the Figure Legends and Methods section.
Randomization	For cell-based assays, cells from the same dish were collected and the same number of cells were seeded randomly into each well of different well-plates for compound/probe treatments.
Blinding	For luciferase-based IFN assay, researchers performing the assay were blind to the identity and nature of the compounds tested (excB was one of many compounds assayed and was labeled with a generic number). Other parts of the study did not involve blinding since it involves chemical proteomics and cell-based assays that require samples to be clearly labeled for data analysis performed by the respective researchers.

Reporting for specific materials, systems and methods

We require information from authors about some types of materials, experimental systems and methods used in many studies. Here, indicate whether each material, system or method listed is relevant to your study. If you are not sure if a list item applies to your research, read the appropriate section before selecting a response.

Materials & experimental systems

n/a	Involved in the study
☐	☒ Antibodies
☐	☒ Eukaryotic cell lines
☒	☐ Palaeontology and archaeology
☒	☐ Animals and other organisms
☒	☐ Clinical data
☒	☐ Dual use research of concern

Methods

n/a	Involved in the study
☒	☐ ChIP-seq
☒	☐ Flow cytometry
☒	☐ MRI-based neuroimaging

Antibodies

Antibodies used

Anti-STING (#13647, 1:1000 dilution), anti-phospho-TBK1 (#5483, 1:1000 dilution), anti-TBK1 (#3504, 1:1000 dilution), anti-phospho-IRF3 S396 (#29047, 1:1000 dilution), anti-IRF3 (#4302, 1:1000 dilution), anti-α-tubulin (#2144, 1:2000 dilution) were purchased from Cell Signaling Technology. Anti-eIF2D (#12840-a-AP, 1:2000 dilution) was purchased from Proteintech. Anti-rabbit-HRP (#111-035-003, 1:20000 dilution) were purchased from Jackson ImmunoResearch Laboratories.

Validation

Antibodies used in this study were validated by the respective commercial sources and available information may be obtained from Cell Signaling Technology, Proteintech, Jackson ImmunoResearch Laboratories, respectively.

Eukaryotic cell lines

Policy information about cell lines and Sex and Gender in Research

Cell line source(s)

RAW 264.7, HEK293T, NIH3T3, THP-1, were obtained from ATCC. THP1-Dual™ KI-hSTING-R232 NF-κB-SEAP and IRF-Lucia reporter cells were obtained from Invivogen.

Authentication

No re-authentication of the cell lines was performed prior to use in this study.

Mycoplasma contamination

The cell lines were tested and confirmed to be mycoplasma-free using the MycoAlert® PLUS Mycoplasma Detection kit (Lonza, LT07-710).

Commonly misidentified lines
(See ICLAC register)

No commonly misidentified cell lines were used in this study.
